# Optimal CXCR5 expression during Tfh maturation involves the Bhlhe40-Pou2af1 axis

**DOI:** 10.1016/j.celrep.2025.116470

**Published:** 2025-10-22

**Authors:** Xiaoliang Zhu, Xi Chen, Yaqiang Cao, Chengyu Liu, Zoey J. Kline, Gangqing Hu, Sundar Ganesan, Tibor Z. Veres, Difeng Fang, Shuai Liu, Danping Wei, Hirofumi Shibata, Dominic P. Golec, Hyunwoo Chung, Ronald N. Germain, Pamela L. Schwartzberg, Keji Zhao, Jinfang Zhu

**Affiliations:** 1 Molecular and Cellular Immunoregulation Section, Laboratory of Immune System Biology, National Institute of Allergy and Infectious Diseases, National Institutes of Health, Bethesda, MD 20892, USA; 2 Laboratory of Epigenome Biology, National Heart Lung and Blood Institute, National Institutes of Health, Bethesda, MD 20892, USA; 3 Transgenic Core Facility, National Heart Lung and Blood Institute, Bethesda, MD 20892, USA; 4 Biological Imaging Section, Research Technologies Branch, National Institute of Allergy and Infectious Diseases, National Institutes of Health, Bethesda, MD 20892, USA; 5 Lymphocyte Biology Section, Laboratory of Immune System Biology, National Institute of Allergy and Infectious Diseases, National Institutes of Health, Bethesda, MD 20892, USA; 6 Cell Signaling and Immunity Section, Laboratory of Immune System Biology, National Institute of Allergy and Infectious Diseases, National Institutes of Health, Bethesda, MD, USA; 7 Lead contact

## Abstract

The pair of transcription factors Bcl6-Blimp1 is well known for follicular T helper (Tfh) early cell fate determination; however, the mechanism(s) for late regulation of CXCR5 during Tfh migration into germinal centers (GCs) is still unclear. In this study, we uncovered another pair of transcription factors, Bhlhe40-Pou2af1, that regulate CXCR5 expression. Pou2af1 was specifically expressed in Tfh cells, whereas Bhlhe40 expression was found to be high in non-Tfh cells. Pou2af1 promoted Tfh formation and migration into a GC by upregulating CXCR5 but not Bcl6, while Bhlhe40 repressed this process by inhibiting Pou2af1 expression. RNA sequencing analysis of antigen-specific Tfh cells generated *in vivo* confirmed the role of Bhlhe40-Pou2af1 axis in regulating optimal CXCR5 expression. Thus, the regulation of CXCR5 expression and migration of Tfh cells into a GC involves a transcriptional regulatory circuit consisting of Bhlhe40 and Pou2af1, which does not affect the Bcl6-Blimp1 circuit that determines the Tfh cell fate.

## In brief

The Bcl6-Blimp-1 axis plays a critical role in follicular T helper (Tfh) cell fate determination; however, the regulation of CXCR5 expression during Tfh maturation is still elusive. Zhu et al. report that the Bhlhe40-Pou2af1 pair regulates optimal expression of CXCR5 and Tfh cell migration into germinal centers without affecting Bcl6 induction.

## Graphical Abstract

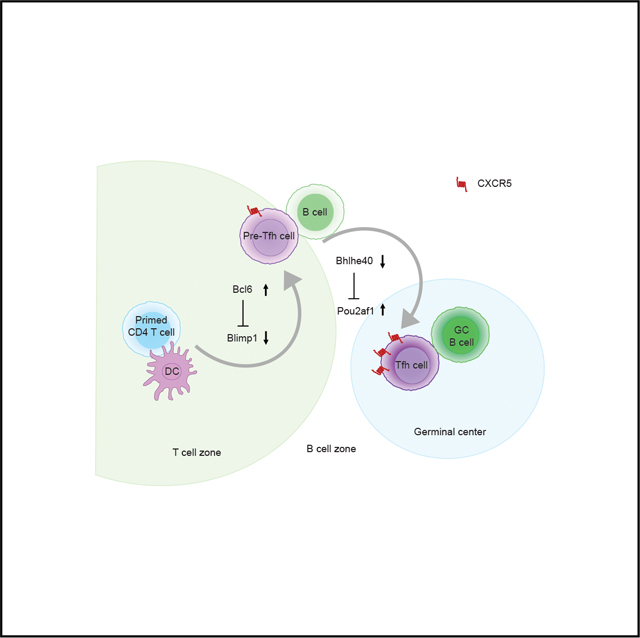

## INTRODUCTION

Follicular T helper (Tfh) cells, as one subset of T helper cells, play a critical role during humoral immune response by helping B cells in antibody production.^1^ Similar to other effector CD4 T (Teff, e.g., Th1/Th2/Th17) cells, Tfh cells differentiate from naive CD4 T cells, and their differentiation is tightly regulated by T cell receptor (TCR) signaling and cytokine milieu. Bcl6 has been identified as the master transcription factor determining the Tfh cell fate, establishing Tfh cells as another distinct lineage of Teff cells.^2–4^

During the early phase of naive CD4 T cells differentiating into pre-Tfh cells induced by antigen presented by dendritic cells (DCs), Bcl6 upregulation determines Tfh cell fate, whereas Blimp1 upregulation results in other Teff cells.^3^ The strength of TCR signaling might play an important role during Tfh and non-Tfh fate determination.^5^ For the second priming, pre-Tfh cells make contact with B cells and receive additional signals from B cells for further development, via ICOS-ICOSL^6^ and SLAM family members.^7^ During the late phase of Tfh cell differentiation, further upregulation of the chemokine receptor CXCR5 and downregulation of the T cell zone-homing chemokine receptor CCR7 are critical for guiding Tfh cell migration into germinal centers (GCs) to become GC-Tfh.^8–10^ In GCs, GC-Tfh exert their functions by helping B cells go through affinity maturation to become high-affinity antibody-secreting plasma cells or long-lived memory B-cells, a process that is important for the host’s defense against various pathogens.^11^

The location of Tfh cells determines their functionality.^12,13^ For example, *Sh2d1a*^−/−^ T cells, although capable of differentiating into early Tfh cells, are unable to be efficiently recruited to and retained in a nascent GC, resulting in an impaired GC reaction.^14^ CXCR5 downregulation and CXCR4 upregulation result in re-distribution of Tfh cells from the light zone to dark zone in the GC, partially explaining the defects of GC responses and poor vaccine-induced immunity in older individuals.^15^ As for Tfh cells, CXCR5 is also essential for B cells located in the light zone of GC,^16^ while CXCR4 is required for B cells migrating into the dark zone of GC.^17^ Although Bcl6 is the master transcription factor for Tfh cell differentiation, it does not directly regulate CXCR5 expression during Tfh cell differentiation, especially during their migration into GCs.^18,19^ Ascl2 initiates Tfh cell differentiation by directly upregulating *Cxcr5* but not *Bcl6*.^20^ However, how Tfh cells maintain and further upregulate CXCR5 expression during pre-Tfh to GC-Tfh is still unknown.

The transcription factor Bhlhe40 (basic-helix-loop-helix [bHLH] family member e40), also known as Bhlhb2, Dec1, or Stra13, is induced by TCR signaling.^21^ Bhlhe40 has been reported to be critical for inducing autoimmune diseases, such as experimental autoimmune encephalomyelitis, by regulating granulocyte-macrophage colony-stimulating factor (GM-CSF) and interleukin (IL)-10.^22^ Through inhibiting IL-10 production, Bhlhe40 helps balance inflammatory and anti-inflammatory type 1 immune responses.^23,24^ Bhlhe40 also regulates the differentiation and function of Th2^25^ and CD8 T cells.^26^ It has been recently reported that Bhlhe40 restrains GC reaction by limiting activated CD4 T cell proliferation and the earliest generation of GC B cells.^27^ However, the mechanism through which Bhlhe40 regulates Tfh cell differentiation is still elusive.

Pou2af1 (POU domain class 2-associating factor 1), also well known as Bob1, OBF-1, or OCA-B, has long been considered a B cell-specific factor that interacts with the octamer transcription factors Oct1 and Oct2 to enhance octamer-dependent transcription.^28^ Pou2af1 has also been reported to be involved in GC formation^29–31^ and Tfh functions.^32,33^ However, what regulates Pou2af1 expression and how Pou2af1 affects Tfh cell function are still unknown.

In this study, we found that Bhlhe40 suppressed Pou2af1 expression during T cell activation toward all Th cell fates. In the absence of Bhlhe40, Tfh cell differentiation was enhanced in a manner dependent on the upregulation of Pou2af1. Pou2af1 was critical for Tfh cell differentiation and migration into GC through upregulation of CXCR5 expression in a T cell-intrinsic manner. On the other hand, Pou2af1 deficiency did not affect initial Bcl6 induction during early Tfh cell differentiation. Thus, our work highlights the importance of the Bhlhe40-Pou2af1 axis downstream of Bcl6-Blimp1 in regulating CXCR5 expression during cell maturation from pre-Tfh to GC-Tfh cells.

## RESULTS

### Bhlhe40 represses Pou2af1 during T cell differentiation

**Figure 1.**
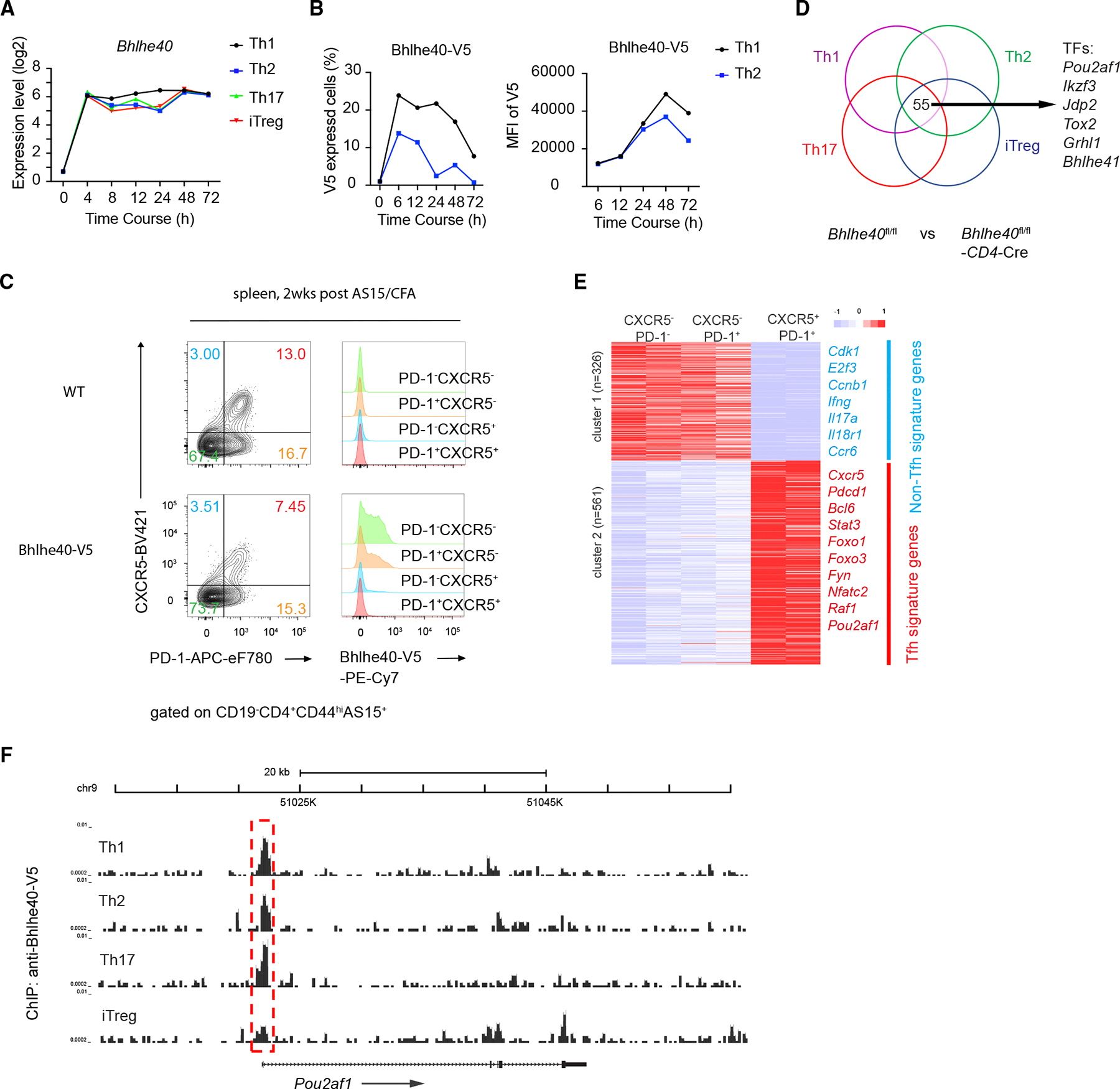
Bhlhe40 represses Pou2af1 in conventional T cells (A) RNA-seq data^34^ were re-analyzed for *Bhlhe40* expression during Th1, Th2, Th17, and iTreg culture *in vitro*. (B) Naive CD4 T cells were purified from Bhlhe40-V5 tagged mice and primed under Th1 or Th2 conditions. Bhlhe40 expression was assessed by anti-V5 staining at the indicated time points. (C) WT and Bhlhe40-V5 tagged mice were immunized (subcutaneously [s.c.]) with AS15/CFA for 2 weeks (2wks), and the expression of *Bhlhe40* by the splenic AS15-specific effector CD4 T cells in four quadrants (PD-1^−^CXCR5^−^, PD-1^+^CXCR5^−^, PD-1^−^CXCR5^+^, and PD-1^+^CXCR5^+^) was assessed by flow cytometry. (D) Naive CD4 T cells from *Bhlhe40*^fl/fl^ or *Bhlhe40*^fl/fl^-CD4Cre mice were cultured under Th1, Th2, Th17, and iTreg conditions, and then harvested for RNA-seq analysis. (E) AS15-specific WT CD4 T cells (B220^−^CD4^+^CD44^hi^AS15^+^) were sorted for three populations (PD-1^−^CXCR5^−^, PD-1^+^CXCR5^−^, and PD-1^+^CXCR5^+^) from inguinal lymph nodes and subjected to RNA-seq analyses. Samples were in biological duplicates. (F) Naive CD4 T cells from Bhlhe40-V5 tagged mice were cultured under Th1, Th2, Th17, and iTreg conditions, and then harvested for ChIP-seq analysis using anti-V5. Samples (D)–(F) were in biological duplicates. Data are representative of two (B and C) independent experiments. See also Figure S1.

We and others have previously found that Bhlhe40 is expressed and functionally important for different types of T helper cells. To further evaluate Bhlhe40 expression, we re-analyzed our previously published RNA sequencing (RNA-seq) results^34^ and found that *Bhlhe40* expression was quickly induced under all four *in vitro* polarization conditions for generating Th1, Th2, Th17, and induced regulatory T (iTreg) cells (Figure 1A), similar to *Irf4* expression (Figure S1A). To assess whether Bhlhe40 is upregulated at the protein level after TCR stimulation, we generated a V5-tagged Bhlhe40 mouse strain (Figure S1B), in which the DNA sequence encoding the V5 tag (GKPIPNPLLGLDST) was inserted into exon 5 of Bhlhe40 immediately before the stop codon. Naive CD4 T cells from these mice were then differentiated under Th1 or Th2 conditions *in vitro* as previously described.^35^ Anti-V5 staining indicated that Bhlhe40 expression at the protein level was also quickly induced by 6 h after anti-CD3 and anti-CD28 stimulation under both Th1 and Th2 conditions (Figures 1B and S1C). Due to the difficulty in generating Tfh cells *in vitro*, we measured Bhlhe40 expression in Tfh cells *in vivo* by immunizing wild-type (WT) and V5-tagged mice with a *Toxoplasma gondii* peptide AS15 together with complete Freund’s adjuvant (CFA) for 2 weeks. Flow cytometry analyses of splenocytes from the immunized mice showed that among the antigen-specific CD4 T (CD19^−^CD4^+^CD44^hi^AS15^+^) cells, Bhlhe40 was highly expressed in non-Tfh (PD-1^−^CXCR5^−^) cells but not in Tfh (PD-1^+^CXCR5^+^) cells (Figure 1C). A similar expression pattern of Bhlhe40 was noted in CD4 T cells harvested from the draining lymph nodes (Figure S1D).

To identify genes that are regulated by Bhlhe40 in distinct subclasses of Th cells, we compared RNA-seq analyses of *Bhlhe40*^fl/fl^ cells with *Bhlhe40*^fl/fl^-CD4Cre cells differentiated under four culture conditions (Th1, Th2, Th17, and iTreg). Fifty-five genes were upregulated in the absence of Bhlhe40 in all four T helper subsets (Figure 1D). Among these genes, six encode transcription factors, with *Pou2af1* ranked at the top. Pou2af1 was reported to be essential for GC formation^29–31^ and required for Tfh cell differentiation and function.^32,33^ Indeed, through RNA-seq analyses of Tfh cells generated *in vivo*, Pou2af1 was found to be highly expressed in the PD-1^+^CXCR5^+^ population with the expression of many other Tfh signature genes (cluster 2) but not non-Tfh signature genes (cluster 1, Figure 1E).

To test whether Bhlhe40 can directly bind to the *Pou2af1* gene, we performed chromatin immunoprecipitation sequencing (ChIP-seq) analysis of Bhlhe40 DNA binding with anti-V5 immunoprecipitation, using *in vitro* polarized Th cells. We found that Bhlhe40 indeed bound to the promoter of *Pou2af1* in all four Th subsets (Figure 1F). Taken together, our results indicate that Bhlhe40 may repress *Pou2af1* expression in T helper subsets through direct binding to its promoter. Given the opposite expression pattern of Bhlhe40 and Pou2af1 in Tfh and non-Tfh, we hypothesized that the Bhlhe40-Pou2af1 axis may play an important role during Tfh cell differentiation.

### Pou2af1 is required for Tfh cell differentiation

**Figure 2.**
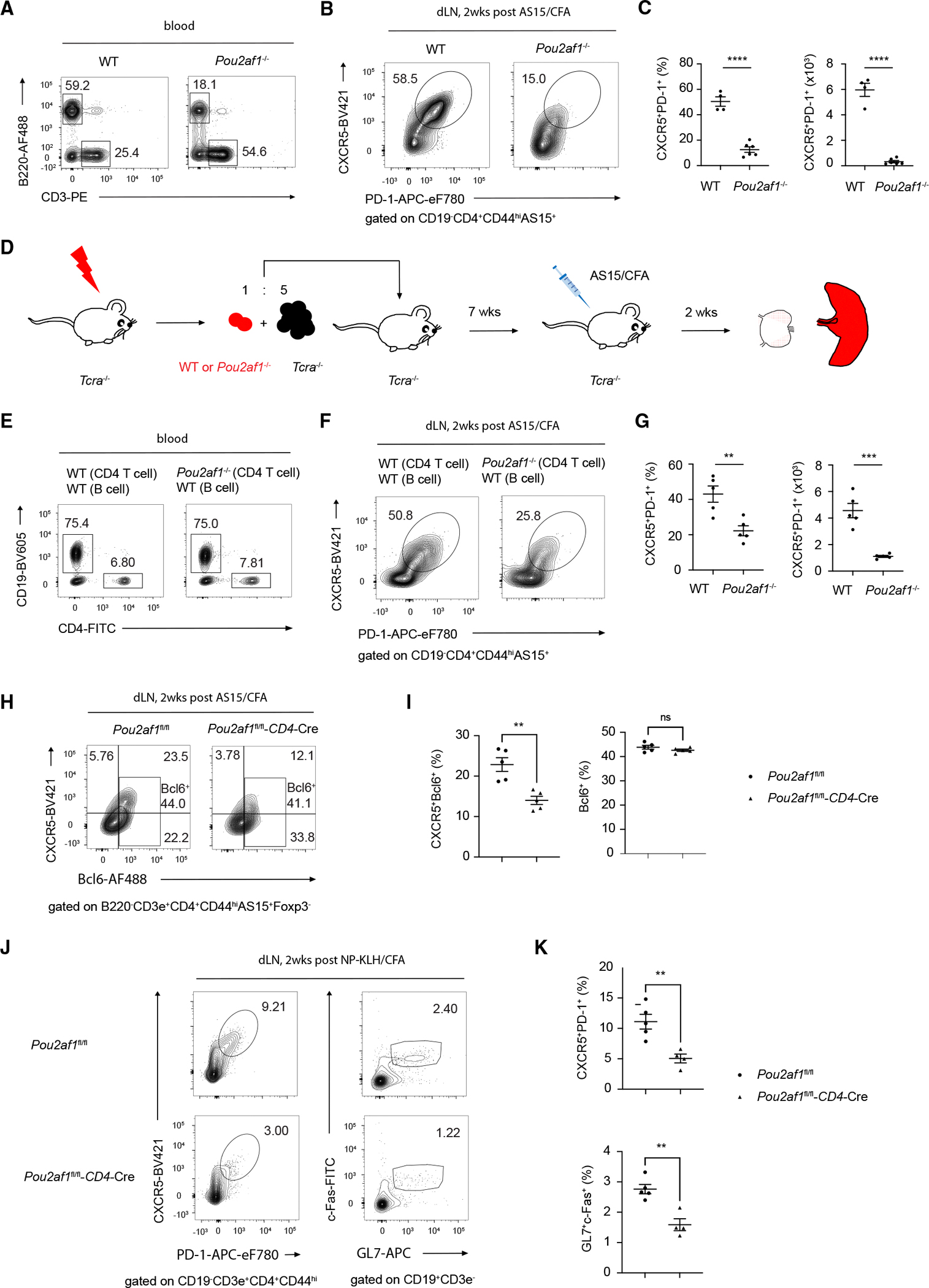
Pou2af1 is required for Tfh cell differentiation (A) Blood cells from WT or *Pou2af1*^−/−^ mice were stained for T cells (B220^−^CD3^+^) and B cells (B220^+^CD3^−^) by flow cytometry. (B) WT and *Pou2af1*^−/−^ mice were immunized (s.c.) with AS15/CFA for 2 weeks, and AS15-specific CD4 T cells (CD19^−^CD4^+^CD44^hi^AS15^+^) from inguinal lymph nodes were analyzed for Tfh (PD-1^+^CXCR5^+^) cells. (C) Summary of differences in percentage and cell numbers of Tfh cells between WT (*n* = 4) and *Pou2af1*^−/−^ (*n* = 6) in (B). (D) Experimental procedure of immunizing BM chimeras *TCRα*^−/−^ mice adoptively transferred a BM mixture of *TCRα*^−/−^ and WT or *Pou2af1*^−/−^ mice with AS15/CFA. (E) Blood cells from (D) were assessed for CD4 T cells (CD19^−^CD4^+^) and B cells (CD19^+^CD4^−^) by flow cytometry. (F) Tfh cells in the inguinal lymph nodes from (D) were analyzed as in (B). (G) Summary of percentage and cell number of Tfh cells from the WT group (*n* = 5) and *Pou2af1*^−/−^ group (*n* = 5) in (D). (H and I) *Pou2af1*^fl/fl^ and *Pou2af1*^fl/fl^-CD4Cre mice were immunized (s.c.) with AS15/CFA for 2 weeks, and cells from inguinal lymph nodes were harvested and analyzed. (H) AS15-specific Foxp3^neg^ cells in inguinal lymph nodes were gated for Tfh (CXCR5^+^Bcl6^+^) and total Bcl6^+^ cells by FACS. (I) Summary of differences in percentage of Tfh cells and total Bcl6^+^ between *Pou2af1*^fl/fl^ group (*n* = 5) and *Pou2af1*^fl/fl^-CD4Cre group (*n* = 5) in (H). (J and K) *Pou2af1*^fl/fl^ and *Pou2af1*^fl/fl^-CD4Cre mice were immunized (s.c.) with NP-KLH/CFA for 2 weeks, and cells from inguinal lymph nodes were harvested and analyzed. (J) Tfh cells (PD-1^+^CXCR5^+^) and GC B cells (GL7^+^c-FAS^+^) in inguinal lymph nodes were analyzed by FACS. (K) Summary of percentage of Tfh cells and GC B cells from *Pou2af1*^fl/fl^ group (*n* = 5) and *Pou2af1*^fl/fl^-CD4Cre group (*n* = 4) in (J). *p < 0.05, **p < 0.01, ***p < 0.001, and ****p < 0.0001, Student’s *t* test. Error bars indicate SEM. Data are representative of more than three (A)–(C) and two (D)–(K) independent experiments. See also Figure S2.

To address the function of Pou2af1 during Tfh cell differentiation, we generated a *Pou2af1* knockout mouse strain (*Pou2af1*^−/−^) by deleting exon3, exon4, and most of exon2 and exon5 as previously described^29^ via CRISPR-Cas9 technology (Figure S2A). Consistent with the previous reports,^30,31^ B cells, but not T cells, in *Pou2af1*^−/−^ mice were found to be reduced as expected (Figures 2A and S2B). We then immunized WT and *Pou2af1*^−/−^ mice with AS15/CFA for 2 weeks and found that Tfh (PD-1^+^CXCR5^+^) cells were reduced in draining lymph nodes of *Pou2af1*^−/−^ mice (Figures 2B and 2C).

Since B cells are known to be involved in Tfh cell generation, to exclude the effect of B cell defect in *Pou2af1*^−/−^ mice, we constructed bone marrow (BM) chimeric animals in which the vast majority of B cells were normal, but T cells were from either WT or *Pou2af1*^−/−^ (Figure 2D). Six weeks after BM reconstitution, we confirmed that B cells in different groups were indeed identical (Figures 2E and S2C). However, Tfh cells were still reduced in *Pou2af1*^−/−^ chimeras compared with WT mixed chimeras (Figures 2F and 2G), demonstrating a T cell intrinsic defect in *Pou2af1*^−/−^ cells to become Tfh cells.

To further examine this finding, we generated a T cell-specific Pou2af1-deficient strain (*Pou2af1*^fl/fl^-CD4Cre, Figure S2D). The generation of Tfh (Bcl6^+^CXCR5^+^) cells in response to immunization was defective in these mice compared to the control *Pou2af1*^fl/fl^ mice, particularly for the CXCR5^hi^ population, while the generation of total Bcl6^+^ cells was not affected (Figures 2H and 2I). This defect was also confirmed by 4-hydroxy-3-nitrophenylacetyl hapten conjugated to keyhole limpet hemocyanin (NP-KLH) together with CFA immunization (Figures 2J and 2K). As a result, GC B cells were also found to be reduced in the *Pou2af1*^fl/fl^-CD4Cre mice (Figures 2J and 2K). Although NP-specific IgG production was normal 2 weeks after immunization (Figure S2E), which could be a result of GC-independent antibody responses, antigen-specific IgGs were significantly reduced in the *Pou2af1*^fl/fl^-CD4Cre mice 6 weeks after immunization (Figure S2F).

The proportion of Ki-67^+^ cells was reduced among AS15^+^CXCR5^+^ population in *Pou2af1*^fl/fl^-CD4Cre compared to the same population in the control mice; however, the proportion of Ki-67^+^ cells was much higher among the AS15^+^CXCR5^hi^ subpopulation compared to the AS15^+^CXCR5^int^ cells, and Pou2af1 did not affect cell proliferation in either subset (Figures S2G and S2H). Therefore, the overall reduction in Ki-67^+^ cells in *Pou2af1*^fl/fl^-CD4Cre group is due to reduced CXCR5^hi^ cells. Furthermore, no defects in antigen-specific Th1, Th17, and Treg responses were observed in the *Pou2af1*^fl/fl^-CD4Cre mice (Figures S2I and S2J).

### Bhlhe40 represses Tfh cell differentiation

**Figure 3.**
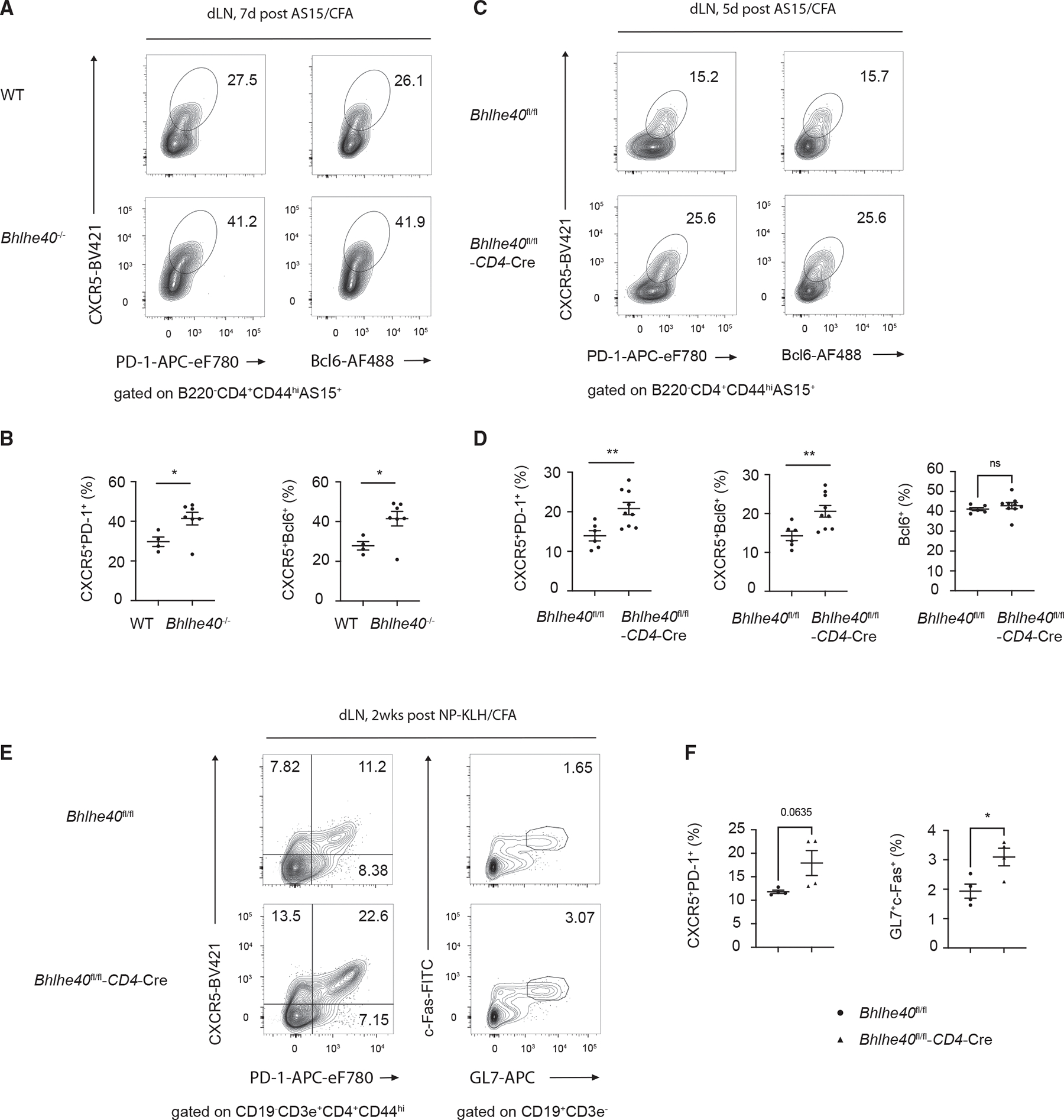
Bhlhe40 represses Tfh cell differentiation (A) WT (*n* = 4) and *Bhlhe40*^−/−^ (*n* = 7) mice were immunized (s.c.) with AS15/CFA for 7 days, and AS15-specific CD4 T cells from inguinal lymph nodes were analyzed for Tfh (PD-1^+^CXCR5^+^ or Bcl6^+^CXCR5^+^) cells by flow cytometry. (B) Summary of percentage difference in Tfh cells between WT and *Bhlhe40*^−/−^ in (A). (C) WT (*n* = 6) and *Bhlhe40*^fl/fl^-CD4Cre (*n* = 9) mice were immunized (s.c.) with AS15/CFA for 5 days, and AS15-specific effective CD4 T cells from inguinal lymph nodes were analyzed for Tfh (PD-1^+^CXCR5^+^ or Bcl6^+^CXCR5^+^) cells by flow cytometry. (D) Summary of percentage difference in Tfh (Bcl6^+^CXCR5^+^) cells and total Bcl6^+^ cells between WT and *Bhlhe40*^fl/fl^-CD4Cre in (C). (E and F) *Bhlhe40*^fl/fl^ and *Bhlhe40*^fl/fl^-CD4Cre mice were immunized (s.c.) with NP-KLH/CFA for 2 weeks, and cells from inguinal lymph nodes were harvested and analyzed. (E) Tfh cells (PD-1^+^CXCR5^+^) and GC B cells (GL7^+^c-FAS^+^) in inguinal lymph nodes were analyzed by FACS. (F) Summary of percentage of Tfh cells and GC B cells from *Bhlhe40*^fl/fl^ group (*n* = 4) and *Bhlhe40*^fl/fl^-CD4Cre group (*n* = 4) in (E). *p < 0.05, **p < 0.01, ***p < 0.001, and ****p < 0.0001, Student’s *t* test. Error bars indicate SEM. Data are representative of two independent experiments. See also Figure S3.

Given that Bhlhe40 represses Pou2af1 and that Pou2af1 is required for Tfh cell differentiation, Bhlhe40 could be involved in repressing Tfh cell formation. Indeed, 1 week after immunization with AS15/CFA, we found that AS15-specific Tfh (PD-1^+^CXCR5^+^ or Bcl6^+^CXCR5^+^) cells were increased in both draining lymph nodes (Figures 3A and 3B) and spleen (Figure S3A) from the Bhlhe40 knockout mice.

To exclude Bhlhe40 function in other types of cells, we performed similar experiments with *Bhlhe40*^fl/fl^-CD4Cre mice, which lack Bhlhe40 expression in T cells. Bcl6^+^CXCR5^+^ cells were increased in *Bhlhe40*^fl/fl^-CD4Cre mice compared with WT mice 5 days post immunization (Figures 3C and 3D). However, total Bcl6^+^ antigen-specific cells did not change in the absence of Bhlhe40 (Figure 3D). After 2 weeks, the difference in Tfh cells between immunized WT and *Bhlhe40*^fl/fl^-CD4Cre became smaller (Figures S3B and S3C), which may be explained by the fact that Bhlhe40 is eventually downregulated during Tfh cell differentiation in WT mice. Nevertheless, GC B cells were significantly increased in the *Bhlhe40*^fl/fl^-CD4Cre mice 2 weeks after immunization (Figures 3E and 3F).

### Mutually exclusive expression of Bhlhe40 and CXCR5 during T cell response *in vivo*

**Figure 4.**
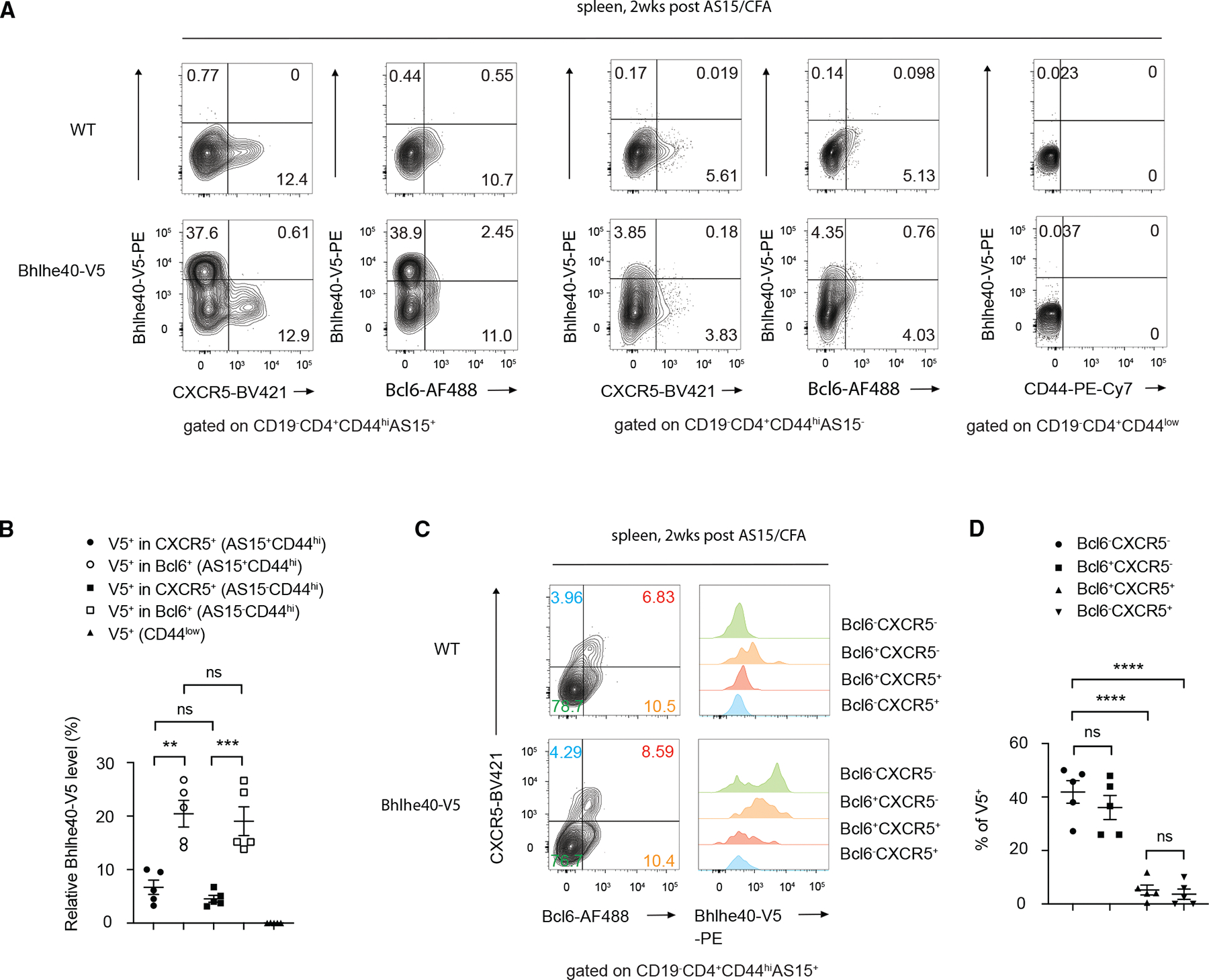
Bhlhe40 and CXCR5 expression is mutually exclusive (A–D) WT and Bhlhe40-V5 mice were immunized (s.c.) with AS15/CFA for 2 weeks, and the splenic AS15-specific CD4 T cells were assessed by flow cytometry. (A) Flow cytometry plots showing the Bhlhe40-V5 expression in AS15^+^ effective CD4 T cells or AS15-effective CD4 T cells or naive CD4 T cells. (B) Summary of percentage differences of relative Bhlhe40-V5 level as indicated in (A). (C) The expression of Bhlhe40 in four subpopulations (Bcl6^−^CXCR5^−^, Bcl6^+^CXCR5^−^, Bcl6^+^CXCR5^+^, and Bcl6^−^CXCR5^+^) was assessed as indicated. (D) Summary of percentage differences of Bhlhe40-V5 as indicated in (C). *p < 0.05, **p < 0.01, ***p < 0.001, and ****p < 0.0001, Student’s *t* test. Error bars indicate SEM. Data are representative of three independent experiments. See also Figure S4.

To further assess the expression levels of *Bhlhe40* during Tfh cell differentiation *in vivo*, we immunized Bhlhe40-V5 mice and then stained CD4 T cells from the spleen using anti-V5, which reflects Bhlhe40 expression. Within the antigen-specific CD4 T (CD19^−^CD4^+^CD44^hi^AS15^+^) cells, Bhlhe40 and CXCR5 showed a mutually exclusive expression pattern, i.e., very few CXCR5^+^V5^+^ cells (Figure 4A). On the other hand, a larger Bcl6^+^V5^+^ population was detected. Overall, the proportion of V5^+^ cells among Bcl6^+^ cells (whether they were AS15-specific or not) was higher than that among the CXCR5^+^ population (Figure 4B). Furthermore, Bhlhe40 expression was detected at a similar level in Bcl6^+^CXCR5^−^ and Bcl6^−^CXCR5^−^ cells, whereas Bcl6^+^CXCR5^+^ and Bcl6^−^CXCR5^+^ cells barely expressed Bhlhe40 (Figures 4C and 4D). These results indicate that Bhlhe40 may play an important role in limiting CXCR5 expression without affecting the Bcl6-Blimp1 axis.

To facilitate studying the expression and functions of Pou2af1, we generated Pou2af1-HA-tdTomato reporter mouse strain through CRISPR-Cas9. In these mice, tdTomato can reflect Pou2af1 expression, whereas the hemagglutinin (HA)-tag on the N-terminal of Pou2af1 can be used to pull down Pou2af1 (Figure S4A). Using these reporter mice, we found that Pou2af1 was highly expressed on a subset of B cells, as expected (Figure S4B), which is also consistent with a previous report.^28^ A portion of B cells from Pou2af1-HA-tdTomato reporter mice (Pou2af1-HA-TG) expressed tdTomato, and the proportion was higher in CXCR5^+^ B cells (36%) than that in CXCR5^−^ B cells (8.3%) (Figure S4B). Upon immunization, we found only a small proportion of antigen-specific CD4 T cells expressed this reporter, indicating that Pou2af1 expression in Tfh cells may be transient and/or dynamic. Nevertheless, Pou2af1-HA-tdTomato was largely enriched among the CXCR5^+^ cells (Figure S4C).

We also bred these reporter mice to *Bhlhe40*^fl/fl^-CD4Cre mice to generate Pou2af1-HA-tdTomato-*Bhlhe40*^fl/fl^-CD4Cre mice. In the absence of Bhlhe40, expression of Pou2af1-HA-tdTomato was increased in cells cultured under Th1 conditions (Figures S4D and S4E) and in activated antigen-specific CD4 T cells in response to immunization *in vivo* (Figures S4F and S4G).

### Pou2af1- and Bhlhe40-mediated gene regulation during Tfh cell differentiation

To gain more insights into how Bhlhe40 and Pou2af1 regulate Tfh cell differentiation, we performed RNA-seq as shown in Figure S5A. By adoptively transferring BM from WT, *Pou2af1*^−/−^, or *Bhlhe40*^−/−^ mice together with that from *TCRα*^−/−^ mice at 1:5 ratio and then immunizing the mice, we were able to study the T cell intrinsic effect of Pou2af1 and Bhlhe40 during Tfh cell differentiation 2 weeks post-immunization. Three sub-populations (PD-1^−^CXCR5^−^, PD-1^−^CXCR5^+^, and PD-1^+^CXCR5^+^) among the antigen-specific CD4 T cells were sorted and subjected to RNA-seq analyses (Figure S5B). Initial analysis of the RNA-seq results confirmed expected genetic deletions in cells from different groups (Figure S5C).

**Figure 5.**
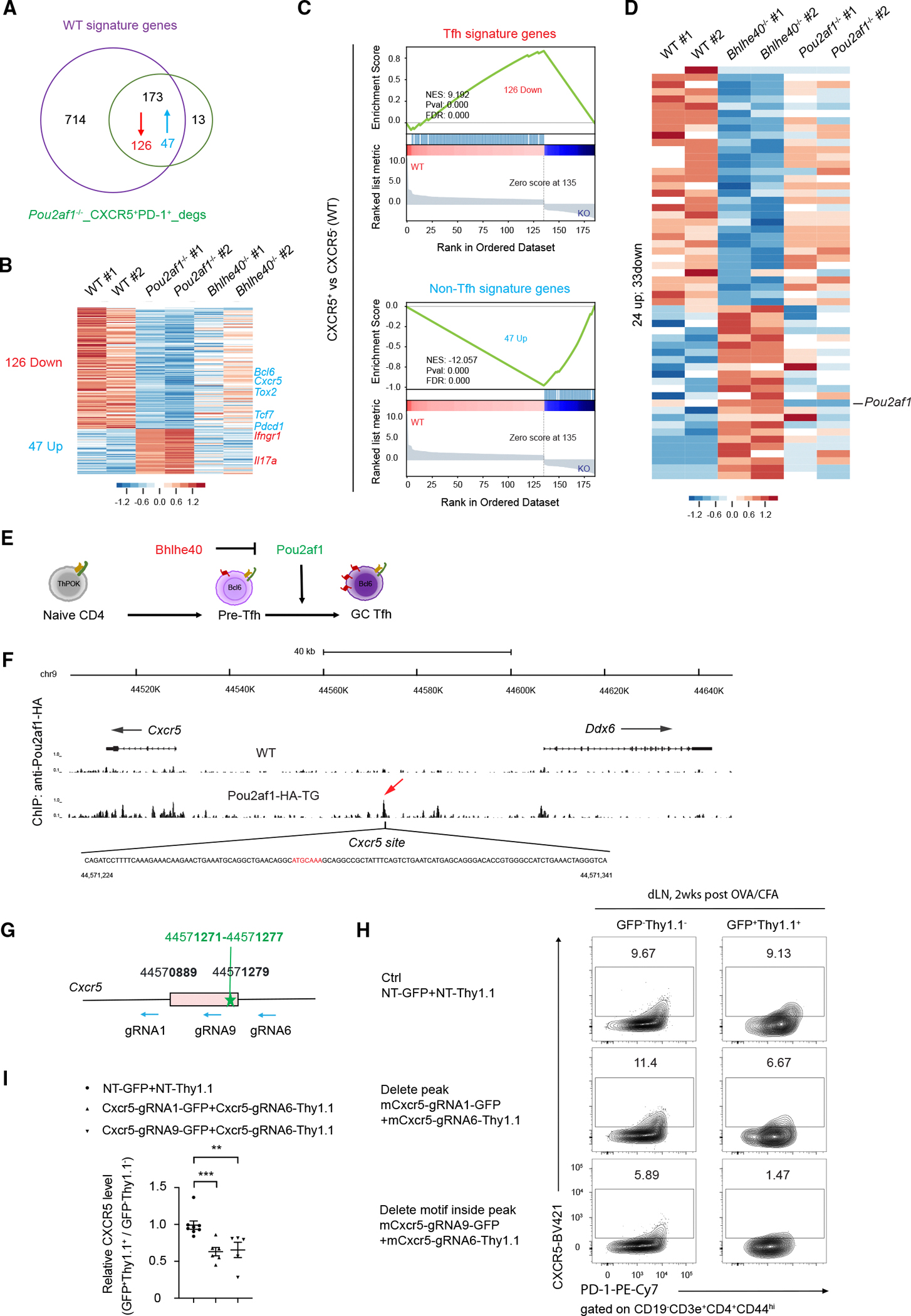
Pou2af1 and Bhlhe40 regulate Tfh cell differentiation in cell-intrinsic manner (A–D) AS15-specific WT, *Pou2af1*^−/−^, and *Bhlhe40*^−/−^ CD4 T cells (B220^−^CD4^+^CD44^hi^AS15^+^) were sorted for three populations (PD-1^−^CXCR5^−^, PD-1^+^CXCR5^−^, and PD-1^+^CXCR5^+^) from inguinal lymph nodes and used for RNA-seq analysis. Samples are in biological duplicates. The experimental procedure was shown in Figure S5A. (A) Overlap between signature genes shown in Figure 1E and DEGs between WT and *Pou2af1*^−/−^ CXCR5^+^PD-1^+^ cells. (B) Heatmap of 126 downregulated genes and 47 upregulated genes among the DEGs in (A). (C) GSEA of the 126 downregulated genes and 47 upregulated genes in (B). (D) DEGs between WT and *Bhlhe40*^−/−^ CXCR5^−^PD-1^+^ cells and their expression in *Pou2af1*^−/−^ CXCR5^−^PD-1^+^ cells. (E) A model for Bhlhe40-and Pou2af1-mediated regulation of Tfh cell differentiation. (F) Splenic B cells were sorted from WT or Pou2af1-HA-TG mice, and anti-HA was used for ChIP-seq analysis of Pou2af1 binding in the genome. An Oct1/2 binding octamer DNA motif^28,36^ (red) was found in *Cxcr5* site. (G) Three gRNAs targeting the Pou2af1-binding site on *Cxcr5* were designed as indicated. Pink box shows Pou2af1-binding peak found in (F), and green star shows Oct1/2-binding octamer DNA motif. (H) Naive CD4 T cells from OTII-Cas9 mice were cultured under Tneutral conditions, infected with the indicated combination of virus, and then adoptively transferred into *Tcra*^−/−^ mice. Transferred CD4 T cells from inguinal lymph nodes after OVA/CFA immunization were analyzed by comparing CXCR5^+^ cells among two populations (WT: Thy1.1^−^GFP^−^; KD: Thy1.1^+^GFP^+^) in the same mice. (I) Summary of relative difference in CXCR5 expression by multiplying the percentage and MFI of CXCR5 in the three indicated groups. *n* = 5–8. *p < 0.05, **p < 0.01, ***p < 0.001, and ****p < 0.0001, Student’s *t* test. Error bars indicate SEM. Samples (A)–(F) were in biological duplicates, and data in (H) and (I) are representative of three independent experiments. See also Figures S5 and S6.

As shown in Figure 1E, we first identified 887 different expression genes (DEGs) within the WT groups (including Tfh signature genes and non-Tfh signature genes), which distinguish three populations of antigen-specific effector CD4 T cell populations (PD-1^−^CXCR5^−^, PD-1^+^CXCR5^−^, and PD-1^+^CXCR5^+^). Comparing WT and *Pou2af1*^−/−^PD-1^+^CXCR5^+^ groups, 186 DEGs were identified, among which 173 overlapped with the 887 signature genes (Figure 5A). CXCR5 was found to be reduced in *Pou2af1*^−/−^ compared with WT (Figure 5B). Strikingly, all 126 downregulated genes in the absence of Pou2af1 were Tfh signature genes, whereas all 47 upregulated genes were non-Tfh-signature genes (Figure 5C). By contrast, genes that are regulated by Pou2af1 were largely not affected by Bhlhe40 deficiency in PD-1^+^CXCR5^+^ cells (Figure 5B), consistent with the fact that Bhlhe40 is not expressed by these cells.

Since several Tfh signature genes, including *Pdcd1* and *Bcl6*, increase expression when Tfh cells move into GCs and receive activation signals from B cells, differences in the expression of these genes between WT and *Pou2af1*^−/−^ PD-1^+^ CXCR5^+^ cells could be due to an indirect effect of CXCR5 regulation by Pou2af1. We therefore asked whether retroviral infection of CXCR5 can rescue the defect of *Pou2af1*^−/−^ cells to become GC-Tfh cells. We first inactivated the *Pou2af1* gene in ovalbumin (OVA)-specific cells using CRISPR-Cas9 with or without CXCR5 overexpression *in vitro* and then adoptively transferred these viral-infected OVA antigen-specific cells into *Tcra*^−/−^ recipients before immunizing with OVA/CFA (Figure S5D). As expected, eliminating the expression of Pou2af1 impaired the generation of PD-1^+^CXCR5^+^ cells, consistent with the observations using other Pou2af1 knockout models (Figures S5E and S5F). Interestingly, retroviral expression of CXCR5 resulted in a restoration of the PD-1^+^CXCR5^+^ cell population, whose generation was reduced in the absence of Pou2af1, especially for the PD-1^hi^CXCR5^hi^ cells (Figures S5E and S5F). Consistent with the results from other Pou2af1-deficient models, Bcl6 expression was not affected by CRISPR-mediated Pou2af1 deletion.

From the RNA-seq analysis, we also found increased Pou2af1 expression in the PD-1^+^CXCR5^−^ population in the absence of Bhlhe40 (Figure 5D). Interestingly, genes that are regulated by Bhlhe40 in this cell subset are not affected by Pou2af1 deletion (Figure 5D), consistent with the fact that these cells express no or low levels of Pou2af1. The dynamic expression of Bhlhe40 and Pou2af1 and their cell context-dependent gene regulation during Tfh cell differentiation support a model that downregulation of Bhlhe40 and thus the induction of Pou2af1 expression allows GC Tfh generation through upregulation of CXCR5 (Figure 5E). To test whether Pou2af1 can directly regulate CXCR5, we performed anti-HA ChIP-seq analysis of B cells harvested from Pou2af1-HA-tdTmato reporter mice. Multiple Pou2af1 binding peaks were identified at the *Cxcr5* locus (Figure S6A). ChIP-seq results also showed that Pou2af1 bound to the *Bcl6* locus, suggesting that while Pou2af1 does not regulate Bcl6 at the protein level, it may directly regulate *Bcl6* transcription (Figure S6B). By contrast, although the expression of *Pdcd1* and *Tox2* was also reduced in the absence of Pou2af1, there was no obvious binding peak at these gene loci (Figures S6C and S6D), suggesting that Pou2af1 indirectly regulates the expression of these genes. We then focus on the highest Pou2af1 binding peak 5′ of the *Cxcr5* gene that contains an Oct1/2 motif (ATGCAAA) (Figure 5F). Indeed, CRISPR-Cas9-mediated deletion of the anti-Pou2af1 ChIP-seq peak or a smaller region containing the Oct1/2 motif reduced CXCR5 expression (Figures 5G–5I).

### Pou2af1 regulates Tfh cell differentiation downstream of Bhlhe40

**Figure 6.**
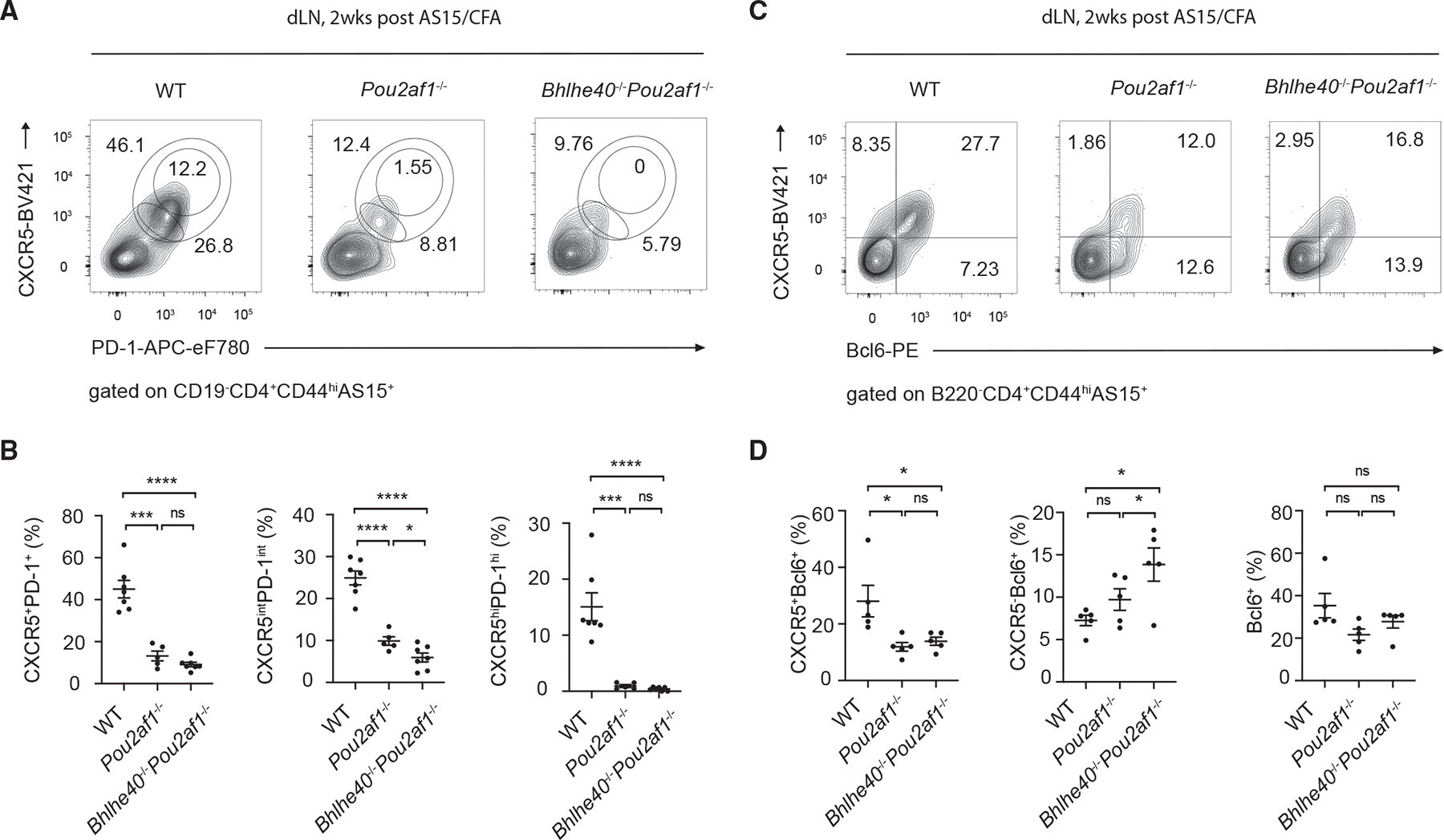
Pou2af1 functions downstream of Bhlhe40 during Tfh cell differentiation (A) WT (*n* = 7), *Pou2af1*^−/−^ (*n* = 5), and *Bhlhe40*^−/−^*Pou2af1*^−/−^ (*n* = 7) mice were immunized (s.c.) with AS15/CFA for 2 weeks, and AS15-specific CD4 T cells from inguinal lymph nodes were analyzed for PD-1^int^CXCR5^int^, PD-1^hi^CXCR5^hi^, and PD-1^+^CXCR5^+^ (including both PD-1^int^CXCR5^int^ and PD-1^hi^CXCR5^hi^) by flow cytometry. (B) Summary of percentage of PD-1^+^CXCR5^+^, PD-1^int^CXCR5^int^, and PD-1^hi^CXCR5^hi^ cells in (A). (C) AS15-specific CD4 T cells from inguinal lymph nodes of WT (*n* = 5), *Pou2af1*^−/−^ (*n* = 5), *Bhlhe40*^−/−^*Pou2af1*^−/−^ (*n* = 5) groups in BM chimeras model as Figure 2E were analyzed for Bcl6^+^CXCR5^+^, Bcl6^+^CXCR5^−^, and Bcl6^+^ (including both Bcl6^+^CXCR5^+^ and Bcl6^+^CXCR5^−^) by flow cytometry. (D) Summary of percentage of Bcl6^+^CXCR5^+^, Bcl6^+^CXCR5^−^, and Bcl6^+^ cells in (C). *p < 0.05, **p < 0.01, ***p < 0.001, and ****p < 0.0001, Student’s *t* test. Error bars indicate SEM. Data are representative of three (A and B) and two (C and D) independent experiments. See also Figure S6.

To confirm the relationship between Pou2af1 and Bhlhe40 in regulating Tfh cell development, we crossed *Pou2af1*^−/−^ mice with *Bhlhe40*^−/−^ mice to generate *Pou2af1*^−/−^*Bhlhe40*^−/−^ mice. By comparing immunized WT, *Pou2af1*^−/−^, and *Pou2af1*^−/−^*Bhlhe40*^−/−^mice, we found that Tfh cells (CXCR5^+^PD-1^+^) were similarly reduced in *Pou2af1*^−/−^*Bhlhe40*^−/−^ mice as in *Pou2af1*^−/−^ mice (Figures 6A and 6B). Since B cells in *Pou2af1*^−/−^*Bhlhe40*^−/−^ mice were also found to be reduced, we performed similar BM chimeras experiments as shown in Figure 2E to exclude the effect of B cell defect. Tfh cells (CXCR5^+^Bcl6^+^) were still reduced in *Pou2af1*^−/−^*Bhlhe40*^−/−^ group (Figures 6C and 6D) while B cells in these WT, *Pou2af1*^−/−^, and *Pou2af1*^−/−^*Bhlhe40*^−/−^ chimeras were similar (Figure S6E). Consistent with the idea that Bhlhe40-Pou2af1 axis mainly regulates CXCR5 expression independent of regulating Bcl6, the Bcl6^+^ population remained similar among WT, *Pou2af1*^−/−^, and *Pou2af1*^−/−^*Bhlhe40*^−/−^ groups, with the accumulation of CXCR5^−^ Bcl6^+^ population when both Bhlhe40 and Pou2af1 were absent (Figures 6C and 6D). Since *Pou2af1*^−/−^*Bhlhe40*^−/−^ largely showed a similar phenotype as *Pou2af1*^−/−^, we conclude that Pou2af1 functions downstream of Bhlhe40 in regulating Tfh cell differentiation.

### Optimal CXCR5 expression is required for Tfh cell migration into GC

**Figure 7.**
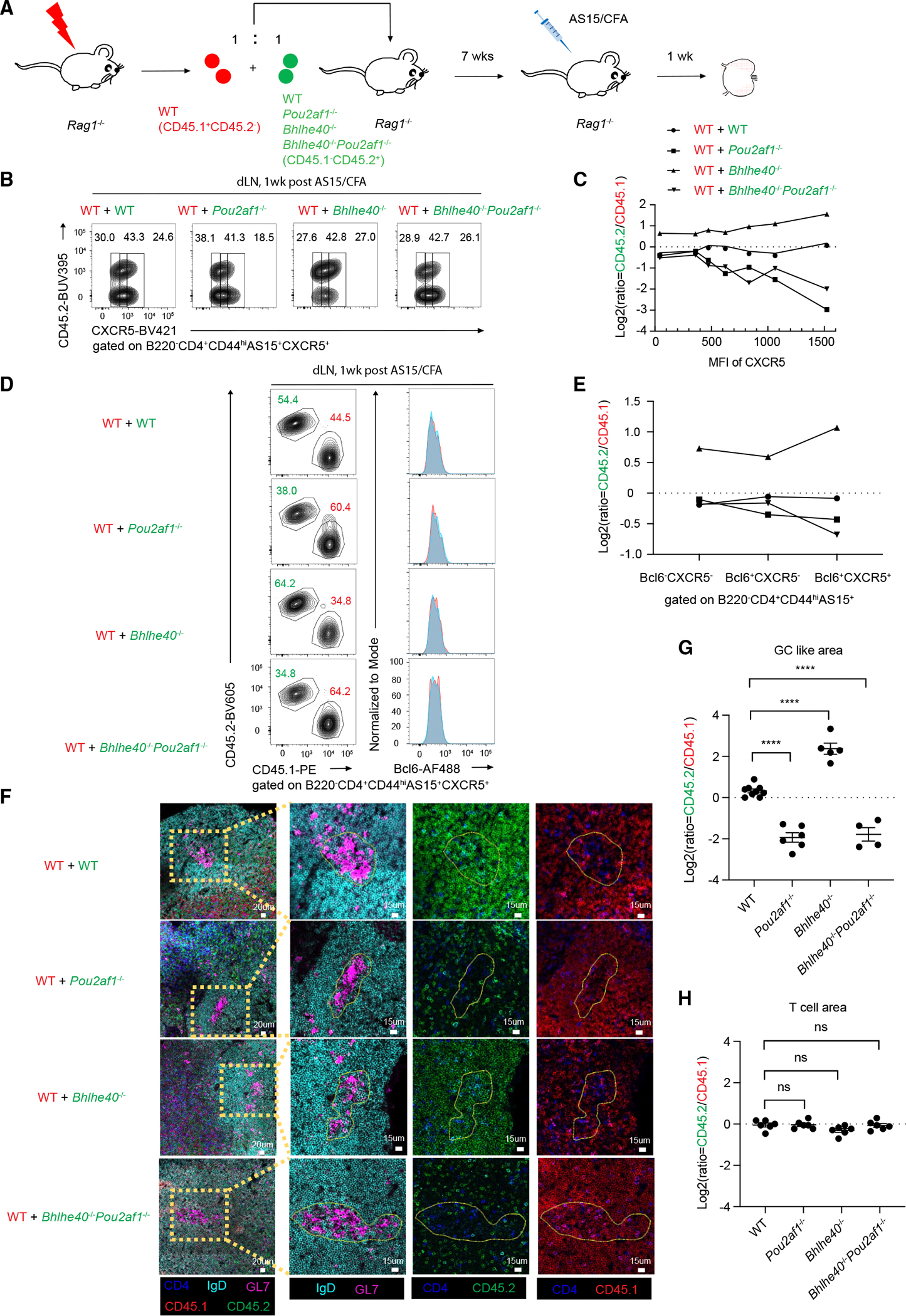
Regulation of optimal CXCR5 level through Bhlhe40-Pou2af1 axis during Tfh cell maturation (A) Experimental procedure of immunizing mixed BM *Rag1*^−/−^ mice by adoptively transferring BM mixture of WT (CD45.1^+^CD45.2^−^) (red) mice with indicated genotyped (WT, *n* = 3; *Pou2af1*^−/−^, *n* = *2*; *Bhlhe40*^−/−^, *n* = *3*; and *Bhlhe40*^−/−^*Pou2af1*^−/−^, *n* = 3) mice (CD45.1^−^CD45.2^+^) (green) mice with AS15/CFA for 1 week.(B) B220^−^CD4^+^CD44^hi^AS15^+^CXCR5^+^ cells from inguinal lymph nodes were assessed by flow cytometry in four different groups from (A). (C) Summary of the cell ratios (indicated genotype/WT in the same mouse) based on different CXCR5 MFIs. (D) Same cells shown in (B) were assessed for Bcl6 expression by flow cytometry. (E) Summary of the cell ratios (indicated genotype/WT in the same mouse) among three cell populations (Bcl6^−^CXCR5^−^, Bcl6^+^CXCR5^−^, and Bcl6^+^CXCR5^+^). (F) Inguinal lymph nodes from (A) were fixed and stained for confocal imaging. Images in yellow boxes on the left were further enlarged to show more details in the GC area (GL7^+^). Anti-CD4 (blue) and anti-IgD (cyan) were used to show the T cell area and B cell area. Anti-GL7 (purple) was used to show the GC. Anti-CD45.2 was used to show different indicated genotyped adoptive cells, and anti-CD45.1 (red) was used to show WT adoptive cells from the same mouse. (G) Summary of ratio of indicated genotyped Tfh cells (CD4^+^CD45.2^+^)/WT Tfh cells (CD4^+^CD45.1^+^) in GC-like area in (F) random GC-like areas were chosen from four indicated genotyped inguinal lymph nodes slice samples (WT, *n* = 9; *Pou2af1*^−/−^, *n* = 6; *Bhlhe40*^−/−^, *n* = 5; and *Bhlhe40*^−/−^*Pou2af1*^−/−^, *n* = 4). (H) Same analysis as in (G) but for T cell area. Random T cell areas were chosen from four indicated genotyped inguinal lymph nodes slice samples (WT, *n* = 6; *Pou2af1*^−/−^, *n* = 6; *Bhlhe40*^−/−^, *n* = 6; and *Bhlhe40*^−/−^ *Pou2af1*^−/−^, *n* = 6). *p < 0.05, **p < 0.01, ***p < 0.001, and ****p < 0.0001, Student’s *t* test. Error bars indicate SEM. Data are representative of two independent experiments. See also Figure S7.

CXCR5 is well-known to be involved in Tfh migration from the T-B border to the GC. Our data have shown that the Bhlhe40-Pou2af1 axis plays an important role in regulating CXCR5 expression independent of Bcl6 regulation. To test whether such optimal regulation of CXCR5 expression by Bhlhe40 and Pou2af1 has any impact on cell migration in a cell-intrinsic manner, we designed another mixed BM experiment in which mixtures of WT BM and BM from different genetic backgrounds were adoptively transferred into irradiated *Rag1*^−/−^ mice (Figure 7A). Seven weeks after BM reconstitution, these *Rag1*^−/−^ recipients were immunized with AS15/CFA. One week later, Tfh cells from the draining lymph nodes were analyzed. As expected, in the “WT (CD45.2^+^) + WT (CD45.2^−^)” group, the mean fluorescence intensity (MFI) of CXCR5 expression by both WT AS15^+^ CD4 T cells was similar (Figure 7B). However, in “*Pou2af1*^−/−^ (CD45.2^+^) + WT (CD45.2^−^)” group, the MFI of CXCR5 was reduced in *Pou2af1*^−/−^ population compared with WT AS15^+^ CD4 T cells in the same animal. By contrast, the MFI of CXCR5 was increased in *Bhlhe40*^−/−^ population compared with WT AS15^+^ CD4 T cells in “*Bhlhe40*^−/−^ (CD45.2^+^) + WT (CD45.2^−^)” group. Furthermore, the MFI of CXCR5 was again decreased in *Pou2af1*^−/−^*Bhlhe40*^−/−^ population compared with the WT counterpart in “*Pou2af1*^−/−^*Bhlhe40*^−/−^ (CD45.2^+^) + WT (CD45.2^−^)” group. There was a progressive reduction in the proportion of *Pou2af1*^−/−^ cells relative to the WT cells with increased CXCR5 expression (Figure 7C). A similar reduction was observed in the *Pou2af1*^−/−^*Bhlhe40*^−/−^ group, reaffirming that Pou2af1 functions downstream of Bhlhe40 in regulating proper CXCR5 expression in a T cell-intrinsic manner.

To test whether Bhlhe40 or Pou2af1 deficiency affects Bcl6 expression in this chimeric model, we further analyzed Bcl6 expression in antigen-specific CD4 T cells from different groups compared with their WT counterparts in the same mice. Consistent with the results shown in Figure 6D, the MFI of Bcl6 expression was similar between WT and gene-deficient cells in the same animal (Figure 7D). Thus, Bhlhe40-Pou2af1 regulates CXCR5 independent of Bcl6 induction. Consequently, an obvious change in the ratio between CD45.1 and CD45.2 populations was observed only when cells progressed from Bcl6^+^CXCR5^−^ to Bcl6^+^CXCR5^+^ in the *Bhlhe40-Pou2af1-*double-deficient chimeras (Figure 7E).

To assess whether optimal expression of CXCR5 has any effect on cell migration to GC, we used these immunized mixed BM chimeric mice for confocal imaging. As shown in Figures 7F and 7G, in “WT (CD45.2^+^) + WT (CD45.2^−^)” group, we found equal numbers of (CD45.2^+^) and (CD45.2^−^) GC-Tfh cells. However, in “*Pou2af1*^−/−^ (CD45.2^+^) + WT (CD45.2^−^)” group, most of the GC-Tfh cells originated from the WT (CD45.2^−^) group. By contrast, *Bhlhe40*^−/−^ (CD45.2^+^) cells were more potent than WT (CD45.2^−^) cells in forming GC-Tfh cells. Again, in “*Pou2af1*^−/−^*Bhlhe40*^−/−^ (CD45.2^+^)+WT (CD45.2^−^)” group, most of the GC-Tfh were from the WT (CD45.2^−^) group. While the ratio of CD45.2/CD45.1 within the GC-Tfh population varied among these chimeras, CD45.2/CD45.1 ratio within CD4 T cells located in the T cell area remained constant (Figures 7H and S7A).

Some CD45.1 WT B cells from all groups developed into GC B cells as expected because of the presence of WT Tfh cells in all the mice (Figures S7B and S7C). However, CD45.1 WT B cells as well as CD45.2 *Bhlhe40*^−/−^ B cells in the “*Bhlhe40*^−/−^ + WT” group developed into GC B cells more efficiently than B cells in other groups, indicating that *Bhlhe40*^−/−^ Tfh cells have a dominant role in promoting GC reactions over the WT Tfh cells. All these data indicate that Bhlhe40 and Pou2af1 modulate Tfh cell migration into the GC in a CD4 T cell-intrinsic manner, which is likely due to effects on CXCR5 expression (Figure S7D).

## DISCUSSION

Tfh cells respond to chemokine CXCL13 through the expression of cell surface marker CXCR5, which is crucial for Tfh migration into the B cell follicle and then GC to become GC-Tfh cells.^8,37,38^ While the Bcl6-Blimp1 axis is critical for Tfh cell differentiation and lineage commitment, Bcl6-independent regulation of CXCR5 expression has been noted. During earlier Tfh cell differentiation primed by DCs, the transcription factor Ascl2 induces CXCR5 expression.^20^ However, what regulates CXCR5 expression at a later stage of Tfh cell maturation is still unknown. Our results here demonstrate an optimal regulation of CXCR5 by the Bhlhe40-Pou2af1 axis during the pre-Tfh to GC-Tfh transition. Bhlhe40 negatively regulates CXCR5 via suppressing Pou2af1 expression. Such regulation is after Tfh cell lineage commitment, which is determined by the Bcl6-Blimp1 axis.

Although through RNA-seq analysis, we noticed that Bcl6 mRNA was reduced in Pou2af1-deficient cell population, which is consistent with previous reports,^32,33^ we were not able to detect the difference in Bcl6 expression by intracellular staining. Similarly, we found several other Tfh signature genes, including *Pdcd1*, reduced in Pou2af1-deficient cell population. These results could be explained by differences in the kinetics of protein and mRNA expression during Tfh cell maturation or further upregulation of these mRNAs when Tfh cells move into the GC and receive additional signals. Indeed, retroviral expression of CXCR5 in Pou2af1-deficient cells was able to restore PD-1 expression, suggesting a possible indirect effect. While it is possible that Pou2af1 may directly regulate Bcl6 expression at the transcriptional level in GC-Tfh cells, our data clearly show that early Bcl6 expression during Tfh cell fate determination is not affected by the Bhlhe40-Pou2af1 axis.

Since Bhlhe40 has been reported to selectively restrict the generation of the earliest GC B cells,^27^ and Pou2af1 is required for GC B cell generation,^39,40^ it is likely that the Bhlhe40-Pou2af1-medicated CXCR5 in B cells may also play an important role in the generation of GC B cells. T-bet, the master transcription factor for Th1 cells, can interact with Bhlhe40 in T cells,^41^ and T-bet can also inhibit Tfh cell differentiation. However, whether T-bet-mediated suppression of Tfh cell differentiation involves Bhlhe40 is unknown.

Bhlhe40 has other functions in T cells, including its role in supporting Treg survival and expansion^42^ and in suppressing IL-10 expression in Th1 cells.^23,24^ Whether Bhlhe40 plays a role in Tfr cells or IL-10 upregulation is involved in Tfh cell differentiation requires further investigation. Nevertheless, our mixed BM chimera experiments indicate that the role of Bhlhe40-Pou2af1 axis in regulating CXCR5 expression and thus GC-Tfh cell generation is cell intrinsic.

In conclusion, we found that the Bhlhe40-Pou2af1 axis plays an important role during Tfh maturation. Bhlhe40 represses Tfh maturation by inhibiting Pou2af1, which regulates optimal expression of the key Tfh migration factor CXCR5. Deficiency in either Bhlhe40 or Pou2af1 does not seem to affect early Tfh cell lineage commitment and Bcl6 induction. Thus, the Bhlhe40-Pou2af1 axis functions after the Bcl6-Blimp1 pair and regulates pre-Tfh cell migration into the GC to become GC-Tfh cells.

### Limitations of the study

Several pathways and molecules have been shown to regulate Bhlhe40 expression: TCR signaling can rapidly induce Bhlhe40 expression; cytokines such as IL-1β can induce Bhlhe40 expression in T cells^43^; GATA3 may regulate Bhlhe40 expression in pathogenic Th17 cells of the experimental autoimmune encephalomyelitis model.^44^ However, we do not know what signal(s) regulate Bhlhe40 dynamics during Tfh cell differentiation. Similarly, while we have shown Bhlhe40 can suppress Pou2af1 expression, what signal(s) induce Pou2af1 expression is unclear. Furthermore, while we observed an important T cell intrinsic function of Pou2af1 in regulating CXCR5 expression, we can only detect a small fraction of Tfh cells expressing Pou2af1 at a given moment. It is likely that Pou2af1 expression in T cells is transient, but its expression in B cells is more stable. Future fate-mapping experiments may clarify this point. Finally, Bhlhe40 and Pou2af1 may regulate gene expression in a cell context-dependent manner. For example, Bhlhe40 inhibits Tox2 expression *in vitro*; however, we did not observe such regulation *in vivo*. The significance of Tox2 regulation by Bhlhe40 requires further investigation. It has been recently reported that after Tfh lineage commitment, there are several sequential developmental stages controlled by Foxp1 and related to IL-21 expression.^45^ However, neither Foxp1 nor IL-21 is regulated by Pou2af1 in our study. It will be important in the future to investigate how the transcription network precisely controls different aspects of Tfh cell development, maturation, and function.

## RESOURCE AVAILABILITY

### Lead contact

Further information and requests for resources and reagents should be directed to and will be fulfilled by the lead contact, Jinfang Zhu (jfzhu@niaid.nih.gov).

### Materials availability

*Bhlhe40*-V5, *Pou2af1*-HA-tdTomato, *Pou2af1*^−/−^, *Pou2af1*^fl/fl^, *Pou2af1*^fl/fl^-CD4Cre, and Cas9-OTII mouse strains are generated in this study.

### Data and code availability

- The RNA-seq and ChIP-seq datasets have been deposited and are available at the Gene Expression Omnibus (GEO) database under the accession number GEO: GSE247137, which is also listed in the key resources table.
- This paper does not report original code.
- Any additional information required to re-analyze the data reported in this paper is available from the lead contact upon request.

## STAR★METHODS

Detailed methods are provided in the online version of this paper and include the following:

### EXPERIMENTAL MODEL AND STUDY PARTICIPANT DETAILS

#### Mice

All the mouse strains are on C57BL/6 background. Wide type (WT) C57BL/6 mice were purchased from Taconic Farms. CD45.1^+^ congenic mice (line 7; Taconic), *Rag1*^−/−^ mice (line 146; Taconic) and *Tcra*^−/−^ (Line 98, Taconic) were from the National Institute of Allergy and Infectious Diseases (NIAID)-Taconic repository. Cas9-OTII mice (line 21272; Taconic) were generated by crossing Cas9 mice (JAX 026179) with OT-II mice (line 187; Taconic). *Bhlhe40*^fl/fl^ mice and *Bhlhe40*^fl/fl^-CD4Cre mice have been previously described.^24^ *Bhlhe40* germline deficient allele was generated by crossing *Bhlhe40*^fl/fl^ mice with EIIa-Cre mice^46^ (JAX 003724), in which the Cre recombinase was expressed at the early mouse embryo stage resulting in germline deletion of loxP-flanked *Bhlhe40* exon 4.

Sperm from *Pou2af1*^*tm1a(KOMP*^*)*^*Wtsi*^ mice on the C57BL/6 background (derived from clone BL5179) was obtained from the University of California, Davis, KOMP Repository. The mouse line was resurrected by *in vitro* fertilization. The *Pou2af1*^+/flox-frt-neo^ mice were genotyped per instructions for the IKMC project 77527 and crossed with the Flpe mice (JAX 005703) to delete the lacZ-loxP-neo-FRT cassette *in vivo.* The resulting *Pou2af1*^fl/fl^ mice were then crossed to *CD4*-Cre transgenic mice (Taconic Line 4196) to generate *Pou2af1*^fl/fl^-CD4Cre. Primers (5′-TACAGAGAGACTAGACACGGTCTGC-3′, 5′-AGAAGGCCTCGTTACACTCCTATGC-3′) were used for PCR to confirm that the lacZ-loxP-neo-FRT was successfully deleted by Flpe. Primers (F/R; 5′-TACAGAGAGACTAGACACGGTCTGC-3′, 5′-GATGAGGACTCTGGGTTCAGAGAGG-3′) were used to PCR to confirm exon 1–3 deletion by Cre.

The following three mouse strains were generated by the CRISPR-Cas9 technology. For Bhlhe40-V5 tagged mice, the single-stranded Bhlhe40-V5 oligonucleotides (CTCTTCGGCCTTGCTCCAGGCTTTGAAGCAGATCCCTCCTTT.

AAACTTAGAAACCAAAGACGGCAAGCCTATCCCCAACCCTCTCCTCGGCCTCGATTCTACCTAAACTCTGGAGGGATCTCCTGCTGCCTTGCTTTCTTTCCTCCCTAATTCCAAAAACCAC), serving as the repair template, and the Bhlhe40-V5 sgRNA (ACCAAAGACTAAACTCTGGAGGG), serving as the single guide RNA (sgRNA) for targeting the *Bhlhe40* locus, were used to generate the Bhlhe40-V5 tagged mouse strain. The primer pair for identifying Bhlhe40-V5 (5′-CAAGATACCGACTCCCTTGCTTC-3′; 5′-GAATCTTCTCTGTGGGTCTGCAG-3′) was used for PCR amplification and the knock-in mutant was confirmed by DNA sequencing. The Bhlhe40-V5 tagged mutant mice were further verified by flow cytometry after staining T cells with anti-V5. To generate the *Pou2af1*^−/−^ mouse strain, two sgRNAs, Pou2af1KO-5′ (ATACCAGGGTGTTCGAGTCAAGG) and Pou2af1KO-3′ (CAACCACACCCTCTCCGTGGAGG), were used to delete exon 2–5 of the *Pou2af1* gene. The primer pair for Pou2af1 (5′-AAGCCCTGTCACTTTTAGACATAGGAGAG-3′; 5′-TGCAGACTTGGTGACATTCCGTATAAAGC-3′) was used for PCR genotyping to verify exon 2–5 deletion which was also confirmed by DNA sequencing. Two founders with exon 2–5 deletion, Pou2af1-2925-#22 (PCR product size 318 bp) and Pou2af1-2923-#20 (PCR product size 567 bp), were chosen and backcrossed with C57BL/6 for more than 6 generations to eliminate a possible off target effect of CRISPR-Cas9, and then intercrossed to generate *Pou2af1*^−/−^ mice. Pou2af1-2923-#20 was crossed with *Bhlhe40*^−/−^ to generate *Bhlhe40*^−/−^*Pou2af1*^−/−^, and other cross mice were all coming from Pou2af1-2925-#22. To generate Pou2af1-HA-tdTomato mouse strain, the single-stranded repair template was generated by inserting the DNA sequence of tdTomato followed by T2A and 3 repeats of HA tag and 10 repeats of Glycine at the ATG translation start site of the coding sequence of *Pou2af1*. The Pou2af1-HA-tdTomato sgRNA was served as the single guide RNA for targeting the *Pou2af1* locus. The primer pair Pou2af1-HA-TdTomato (5′-ACAACAACATGGCCGTCATCAAAGAGTTCA-3′; 5′-TGATGTCCAGCTTGGTGTCCACGTAGTAGTA-3′) was used for PCR and the knock-in mutant was confirmed by DNA sequencing. The Pou2af1-HA-tdTomato mutant mice were further verified by flow cytometry. *Bhlhe40*^fl/fl^ mice or *Bhlhe40*^fl/fl^-*CD4*Cre mice were crossed with Pou2af1-HA-tdTomato mice to generate Pou2af1-tdTomato reporter with or without deletion of *Bhlhe40* in CD4 T cells. Experiments were done when mice were 8–14 weeks of age under protocols approved by the NIAID Animal Care and Use Committee. Both males and females were used, and no differences were noticed. Mice were bred and/or maintained in the NIAID specific pathogen-free animal facility.

### METHOD DETAILS

#### Mouse manipulation

In most immunization experiments, mice received 20 μg AS15 peptide (AVEIHRPVPGTAPPS; synthesized by New England Peptide, Inc.) emulsified in the oil and Complete Freund’s adjuvant (CFA, 2mg/mL, F5881, Sigma) at a 1:1 volume ratio using subcutaneous s.c. injection at two flank sites along the back (50 μL/site). In other experiments, instead of using AS15 peptide, either NP-Keyhole limpet hemocyanin (NP-KLH, 100 μg) or chicken ovalbumin (OVA, 150 μg) emulsified in the oil and CFA was used. After 5–16 d, inguinal draining lymph nodes (dLN) or spleen were harvested to make single-cell suspension for staining or fixed for imaging. To detect AS15-specific CD4 T cells, cells were stained at 37°C for 45min with Tetramer I-Ab-AVEIHRPVPGTAPPS-APC provided by the National Institutes of Health Tetramer Core Facility. In some experiments, serum from blood of the mice immunized for 2 weeks or 6 weeks were collected for ELISA assay.

#### Bone marrow chimeras and cell transfer

For *Tcra*^−/−^ recipient mice, total bone marrow cells recovered from WT, *Pou2af1*^−/−^, *Bhlhe40*^−/−^, or *Bhlhe40*^−/−^*Pou2af1*^−/−^ mice, were mixed with *Tcra*^−/−^ bone marrow cells at a ratio of 1:5, and then adoptively transferred into sub-lethally irradiated (450 rads) *TCRα*^−/−^ mice. For *Rag1*^−/−^ recipient mice, total bone marrow cells recovered from WT CD45.1^+^ congenic mice (line 7; Taconic), were mixed with bone marrow cells from WT, *Pou2af1*^−/−^, *Bhlhe40*^−/−^, or *Bhlhe40*^−/−^*Pou2af1*^−/−^ mice at 1:1 ratio, and then adoptively transferred into sub-lethally irradiated (450 rads) *Rag1*^−/−^ mice. After 6 weeks, reconstitution was confirmed by flow cytometry, and mice were then immunized with AS15/CFA one week later. One or two weeks after immunization, cells from dLN or spleen were harvest for further analysis.

#### Cell culture and purification

Naive CD4 T cells (CD4^+^CD44^lo^CD62L^hi^CD25^−^) were sorted from peripheral lymph nodes by using FACSAria (BD Biosciences Biosciences). Sorted cells were mixed with T cell depleted splenocytes^24^ with a ratio of 1:5 and then cultured under neutral conditions (anti-CD3, 1 μg/mL; anti-CD28, 3 μg/mL; and IL-2, 100 U/mL), Th1 conditions (anti-CD3, 1 μg/mL; anti-CD28, 3 μg/mL; anti–IL-4, 10 μg/mL; IL-12, 10 ng/mL; and IL-2, 100 U/mL), Th2 conditions (anti-CD3, 1 μg/mL; anti-CD28, 3 μg/mL; anti–IFN-γ, 10 μg/mL; IL-12, 10 ng/mL; IL-4, 5000 U/mL; and IL-2, 100 U/mL), Th17 conditions (anti-CD3, 1 μg/mL; anti-CD28, 3 μg/mL; anti–IL-4, 10 μg/mL; anti–IFN-γ, 10 μg/mL; anti–IL-12, 10 μg/mL; TGFβ1, 1 ng/mL; IL-6, 10 ng/mL; and IL-1β, 10 ng/mL), or iTreg conditions (anti-CD3, 1 μg/mL; anti-CD28, 3 μg/mL; anti–IL-4, 10 μg/mL; anti–IFN-γ, 10 μg/mL; anti–IL-12, 10 μg/mL; TGFβ1, 5 ng/mL; and IL-2, 100 U/mL) for 4 d. Antigen specific CD4 T cells (B220^−^CD4^+^CD44^hi^AS15^+^) were FACS-sorted for three populations (PD-1^−^CXCR5^−^, PD-1^+^CXCR5^−^, PD-1^+^CXCR5^+^) from inguinal lymph nodes and used for RNA-Seq analyses. The basic culturing media is the RPMI medium 1640 (Invitrogen) supplemented with 10% FBS (Hyclone), 2 mM L-glutamine (Gibco), 1 mM sodium pyruvate (Gibco), 1× MEM non-essential amino acids (Gibco), 10 mM HEPES (Gibco), 50 μM β-mercaptoethanol (Sigma), 1× Penicillin Streptomycin (Gibco).

#### Retroviral infection of activated CD4 T cells

Single guide RNAs (sgRNAs) targeting various regions of the *Cxcr5* or *Pou2af1* genes were subcloned into an optimized retroviral vector designed to express sgRNA (MSCV-sgRNA-IRES-GFP or MSCV-sgRNA-IRES-Thy1.1).^47^ Sequences of individual sgRNAs are as follows: NT (5′-GCTTCTACTCGCAACGTATT-3′), Cxcr5-gRNA1 (5′-TCCTTTCCAAATATAAGACA-3′), Cxcr5-gRNA6 (5′-CCCTAGTTTCAGATGGCCCA-3′), Cxcr5-gRNA9 (5′-TGAAATGTCAGGTTCCGGTC-3′), Pou2af1KO-gRNA3 (5′-GCGTGGCCATACCAGCGTTG-3′). *Cxcr5* mRNA (NM_007551.3) was cloned into a retroviral expression vector (GFP-RV) that also encodes an IRES-GFP cassette. Primers for cloning *Cxcr5* are as follows: 5′-CTAGGCGCCGGAATTAGATCTATGAACTACCCACTAACCCTG–GACATGGGC-3′, 5′-ATCGATACCGTCGACCTCGAGCTAGAAGGTGGTGAGGGAAGT-AGCATTCTCTGACTC-3’. All cloning was confirmed by Sanger sequencing. Retroviral plasmid and pCL-Eco helper plasmid were transfected into the Phoenix-E cell line using Fugene 6 according to the manufacturer’s instructions, and retroviral supernatant was harvested 48h after transfection. Naive CD4 T cells from Cas9-OTII mice were activated under neutral conditions as described above. 24 h post activation, cells were infected with retrovirus in the presence of polybrene (8 μg/mL final) with spin (3000rpm, 30°) for 60min, and fresh medium was supplied after infection. After another 24h, virus infected cells were harvested and adoptive transfer into recipient mice.

#### Flow cytometry

Single-cell suspensions or stimulated cells were first incubated at 4°C for 15 min with anti-CD16/32 (clone 2.4G2) for blockade of Fc receptors. Cell surface molecules were then stained with various antibodies in PBS with 3% FBS at room temperature for 30 min. For intracellular staining of transcription factors, cells were fixed and permeabilized with Foxp3 staining buffer set (00-5523-00; eBioscience) as manufacturer’s instructions. For antigen-specific (AS15^+^) cell staining, cells were pre-stained with AS15-tetramer-APC at 37°C for 45 min before anti-CD16/32 incubation. Flow cytometry data were collected with LSRFortessa or FACSymphony (BD Biosciences) and the results were analyzed with FlowJo software (Tree Star). Antibodies were purchased from several commercial sources indicated below. Antibodies specific to mouse CD19 (eBio1D3), PD-1 (J43), Foxp3(FJK-16s) and IL-17A (17B7) were purchased from eBioscience; antibodies specific to mouse CD45.1 (A20), CD45.2 (104), B220 (RA3-6B2) and CXCR5 (L138D7) were purchased from Biolegend; and antibodies specific to mouse CD3 (145-2C11), CD4 (GK1.5), CD44 (IM7), Bcl6 (K112-91), Ki67 (B56), T-bet (04–46), ROR γ t (Q31–378) and IFN-γ (XMG1.2) were purchased from BD Biosciences. Fixable Viability Dye eFluor 506 was from eBioscience. AS15-tetramer-APC was provided by the NIH Tetramer Core Facility.

#### Immunofluorescent staining

Bone marrow chimera mice were immunized with AS15/CFA, and dLNs were harvested after 7 d. Samples were treated with 1% fixation buffer (554655; BD Bioscience) at 4°C overnight. Samples were then dehydrated in 30% sucrose and embedded in optimal cutting temperature freezing media (4585, fisher healthcare). CM1950 cryostat (Leica) and Superfrost Plus slides (VWR) were used to make sections, which were further permeabilized and blocked in PBS (0.3% Triton X-100, 1% BSA, 2% normal mouse serum and 2% normal rabbit serum) and then stained with antibodies in the same block buffer. Antibodies against IgD (11-26c.2a), CD4 (GK1.5), and CD45.2 (104) were purchased from Biolegend; antibody against CD45.1 (A20) was purchased from eBioscience; antibody against GL7 (GL7) was purchased from BD Bioscience. Fluoromount G (Southern Biotech) and Leica TCS SP8 confocal microscope were used to collect signaling from slides. Data were analyzed by Imaris software (Bitplane).

#### ELISA

Sera from immunized mice were collected 2 wks or 6 wks after immunization as indicated. Serum samples were analyzed by ELISA for measurement of NP-specific antibodies. Plates were coated overnight with 75ng/mL NP32-BSA in 100 μl of coating buffer (pH = 9.5) at 4°C. After blocking, 1:5000 dilutions of serum were applied to the plates and incubated at room temperature for 2 h. NP-specific IgG1, NP-specific IgG2c and NP-specific total IgG were captured by respectively antibodies (anti-IgG1, anti-IgG2c, and anti-total IgG) for another one hour incubation, followed by anti-(H + L)-HRP for one more hour incubation. After washing, plates were incubated with TMB substrate (Invitrogen) at room temperature for 15 min and stop buffer (invitrogen) was added to stop the reaction. Absorbance was measured at 450nm with a microplate reader (Molecular Devices).

#### RNA-seq, ChIP-seq, and data analysis

For RNA-seq analyses of Th1, Th2, Th17 and iTreg, naive CD4 T cells from *Bhlhe40*^f/f^ or *Bhlhe40*^f/f^-CD4Cre were purified from lymph nodes and primed *in vitro* for 4 d. For RNA-Seq analysis of B220^−^CD4^+^CD44^hi^AS15^+^ cells, three populations (PD-1^−^CXCR5^−^, PD-1^+^CXCR5^−^, PD-1^+^CXCR5^+^) from inguinal lymph nodes were sorted from the indicated bone marrow chimera mice (WT, *Pou2af1*^−/−^, *Bhlhe40*^−/−^). The RNA-Seq was performed as previously described.^48^ Briefly, the total RNAs were harvested and purified by using Qiagen’s miRNeasy micro kit (217084; Qiagen). Qiagen’s DNase set (79254; Qiagen) was used for on column DNA digestion. PolyA-tailed RNAs were purified from total RNA by using Dynabeads mRNA DIR ECT kit (61012; Ambion Life Technologies). The RNA-Seq libraries were sequenced with Illumina HiSeq system, and 50 bp reads were generated by the National Heart, Lung, and Blood Institute DNA Sequencing Core.

For ChIP-seq analysis in Th1, Th2, Th17 and iTreg, naive CD4 T cells from Bhlhe40-V5 tagged mice were purified from lymph nodes and primed *in vitro* for 4 d, and anti-V5 (R96025, Thermofisher) was used for ChIP-Seq analysis of Bhlhe40 binding in the genome. For ChIP-Seq analysis of Pou2af1 binding in the genome, splenic B cells were sorted from WT or Pou2af1-HA-TG mice, and anti-HA (ab9110, Abcam) was used for ChIP. Cells were cross-linked with 1% formaldehyde for 10 min at room temperature and sonicated in the shearing buffer (0.4% SDS in TE buffer) to generate 100 to 500 bp DNA fragments. 10% of total DNA was used as input, and the rest was used in the subsequent immunoprecipitation. DNA-protein complexes were pulled down with specific antibodies in the RIPA buffer overnight. DiaMag anti-mouse IgG coated magnetic beads or DiaMag protein A coated magnetic beads were added into the buffer for additional 4 h. After washing, reverse-crosslinking and DNA purification (100–500 bp) were performed. Purified DNAs were then made into indexed libraries and sequenced.

For RNA-seq and ChIP-seq data analyses, we used the mouse reference genome mm10 and GENCODE (M21)^49^ annotations. We preprocessed all the genomic data generated in this study from raw reads to expression levels or tracks (bigWig files), following the procedures outlined in our previous studies.^50,51^ WashU Epigenome Browser^52^ was used to visualize sequencing data tracks. Specifically, we first used Cuffdiff (v2.2.1)^53^ with default parameters to identify differentially expressed genes (DEGs) between the three wild-type AS15-specific populations (1 vs. others). We used a fold change cutoff ≥2 and a *p*-value cutoff <0.001. All DEGs from the comparison were then clustered using Cluster 3.0^54^ with the following parameters: -cg a -g 7 -m m -k 2 -r 1000. The two clusters of genes were further defined as the Tfh and Non-Tfh signature genes, which were used for the following Gene Set Enrichment Analysis (GSEA)^55^ performed by GSEApy^56^ with the subfunction of prerank and default parameters for DEGs obtained from *Pou2af1*^−/−^ cells.

### QUANTIFICATION AND STATISTICAL ANALYSIS

Samples were compared with Prism 9 software (GraphPad) by a two-tailed unpaired Student’s *t*-test. Data were presented as mean ± SEM. A *p* value <0.05 was considered statistically significant and indicated as *; *p* < 0.01 was indicated as **; *p* < 0.001 was indicated as ***; and *p* < 0.0001 was indicated as ****. Not statistically significant was indicated as n.s.

## Supplementary Material

1

SUPPLEMENTAL INFORMATION

Supplemental information can be found online at https://doi.org/10.1016/j.celrep.2025.116470.

## Figures and Tables

**KEY RESOURCES TABLE:** 

REAGENT or RESOURCE	SOURCE	IDENTIFIER

Antibodies		

Anti-CD3e Alexa Fluor^®^ 700 (500A2)	Biolegend	Cat#152316; RRID: AB_2632713
Anti-CD3e PE (145-2C11)	BD	Cat#553064; RRID: AB_394597
Anti-CD3e FITC (145-2C11)	BD	Cat#553062; RRID: AB_394595
Anti-CD3e Percp (145-2C11)	Biolegend	Cat#100326; RRID: AB_893317
Anti-CD3e eFluor 450 (145-2C11)	eBioscience	Cat#48-0031-82; RRID: AB_10735092
Anti-CD4 BUV395 (GK1.5)	BD	Cat#563790; RRID: AB_2738426
Anti-CD4 APC (RM4-5)	eBioscience	Cat#17-0042-8; RRID: AB_469323
Anti-CD4 FITC (GK1.5)	eBioscience	Cat#11-0041-85; RRID: AB_464893
Anti-CD4-BV650 (GK1.5)	BD	Cat#563232; RRID: AB_2738083
Anti-CD4-BV421 (GK1.5)	Biolegend	Cat# 100438; RRID: AB_11203718
Anti-CD8 Brilliant Violet 605^™^ (53-6.7)	Biolegend	Cat#100744; RRID: AB_2562609
Anti-CD8 APC-eFluor 780 (53-6.7)	eBioscience	Cat#47-0081-82; RRID: AB_1272185
Anti-CD19 BV605 (1D3)	BD	Cat#563148; RRID: AB_2732057
Anti-CD19 APC (1D3)	eBioscience	Cat#17-0193-82; RRID: AB_1659676
Anti-CD19 PerCP-Cy^™^5.5 (1D3)	BD	Cat#551001; RRID: AB_394004
Anti-CD44 APC-eFluor 780 (IM7)	eBioscience	Cat#47-0441-82; RRID: AB_1272244
Anti-CD44 PE-Cy7 (IM7)	BD	Cat#560569; RRID: AB_1727484
Anti-CD44 APC (IM7)	eBioscience	Cat#17-0441-82; RRID: AB_469390
Anti-CD44 BV650 (IM7)	BD	Cat#740455; RRID: AB_2740182
Anti-CD44 BV605 (IM7)	BD	Cat#563058; RRID: AB_2737979
Anti-CD44 BUV737 (IM7)	BD	Cat#612799; RRID: AB_2870126
Anti-CD45.1-PE (A20)	eBioscience	Cat#12-0453-82; RRID: AB_465675
Anti-CD45.2-BUV396 (104)	BD	Cat#564616; RRID: AB_2738867
Anti-CD45.2-BV605 (104)	Biolegend	Cat#109841; RRID: AB_2563485
Anti-CD45.2-FITC (104)	Biolegend	Cat#109806; RRID: AB_313443
Anti-CD279 (PD-I) APC-Cy7 (J43)	eBioscience	Cat# 135224; RRID: AB_2563523
Anti-CD279 (PD-1) PE/Cyanine7 (29F.1A12)	Biolegend	Cat#135216; RRID: AB_10689635
Anti-B220 APC (RA3-6B2)	Biolegend	Cat# 103212; RRID: AB_312997
Anti-B220 Alexa Fluor^®^ 488 (RA3-6B2)	Biolegend	Cat#103225; RRID: AB_389308
Anti-B220 Brilliant Violet 605^™^ (RA3-6B2)	Biolegend	Cat#103244; RRID: AB_2563312
Anti-Bcl6-PE (K112-91)	BD	Cat#561522; RRID: AB_10717126
Anti-Bcl6 Alexa Fluor^®^ 488 (K112-91)	BD	Cat#561524; RRID: AB_10716202
Anti-CD185 (CXCR5) Brilliant Violet 421^™^ (L138D7)	Biolegend	Cat#145512; RRID: AB_2562128
Anti-CD95(Fas) FITC (Jo2)	BD	Cat#554257; RRID: AB_395329
Anti-CD95(Fas) BV650 (Jo2)	BD	Cat#740507; RRID: AB_2740227
Anti-Foxp3 AF700 (FJK-16s)	eBioscience	Cat#56-5773-82; RRID: AB_1210557
Anti-Foxp3 eFluor 450 (FJK-16s)	eBioscience	Cat#48-5773-82; RRID: AB_1518812
Anti-Foxp3 PE (FJK-16s)	eBioscience	Cat#12-5773-82; RRID: AB_465936
Anti-Gata3 Alexa Fluor^®^ 647 (L50-823)	BD	Cat#560068; RRID: AB_1645316
Anti-GL7 Alexa Fluor^®^ 647 (GL7)	BD	Cat#561529; RRID: AB_10716056
Anti-GL7 PE (GL7)	BD	Cat#561530; RRID: AB_10715834
Anti-IFN-γ FITC (Clone XMG1.2)	BD	Cat#554411; RRID: AB_395375
Anti-IL-17A PE-Cy7 (17B7)	eBioscience	Cat#25-7177-82; RRID: AB_10732356
Anti-IgD Alexa Fluor^®^ 700 (11-26c.2a)	Biolegend	Cat#405730; RRID: AB_2563341
Anti-Ki67 BV605 (B56)	BD	Cat#567122; RRID: AB_2916454
Anti-Ki67 PE-Cy^™^7 (B56)	BD	Cat#561283; RRID: AB_10716060
Anti-RoRgt PE (Q31-378)	BD	Cat#562607; RRID: AB_11153137
Anti-T-bet Horizon^™^ V450 (04-46)	BD	Cat#563318; RRID: AB_2687543
Anti-CD90.1 (Thy-1.1) APC-eFluor^™^ 780 (HIS51)	eBioscience	Cat#47-0900-82; RRID: AB_1272252
Anti-CD90.1 (Thy-1.1) Brilliant Violet 605 (OX-7)	Biolegend	Cat#202537; RRID: AB_2562644
Anti-V5-PE (TCM5)	Invitrogen	Cat#12-6796-42; RRID: AB_2784630
Anti-V5-PE-Cy7 (TCM5)	Invitrogen	Cat#25-6796-42; RRID: AB_2784669
Anti-CD3e (2C11)	Harlan	Lot#A2022931
Anti-CD28 (37.51)	Harlan	Lot#A2022929
Anti-IFN-γ (XMG1.2)	Harlan	Lot#A2022930
Anti-IL-4 (11B.11)	Harlan	Lot#A2022930
Anti-CD16/CD32 (2.4G2)	Harlan	Lot#A2083015
Anti-V5 (SV5-Pk1)	Invitrogen	R96025; RRID: AB_2556564
Anti-HA (polyclonal)	Abcam	ab9110; RRID: AB_307019
Chemicals, peptides, and recombinant proteins		
Peptide: AS15 (AVEIHRPVPGTAPPS)	WatsonBio	Order #110911
AS15-Tetramer-APC	NIH TCF	Order#42413
NP-KLH	Biosearch technologies	Cat#*N*-5060-25
NP2-BSA	Biosearch technologies	Cat#*N*-5050X-100
NP32-BSA	Biosearch technologies	Cat#*N*-5050H-100
Albumin from chicken egg white (OVA)	Sigma-Aldrich	Cat#A5503
Freund’s Adjuvant, Complete	Sigma-Aldrich	Cat#F5881-10X10ML
RPMI medium 1640	Gibco	Cat#21870-076
L-glutamine	Gibco	Cat#25030
sodium pyruvate 100mM	Gibco	Cat#11360070
MEM Non-Essential Amino Acids Solution (100×)	Gibco	Cat#111-40-050
HEPES 1M	Gibco	Cat#15630-080
β-mercaptoethanol	Thermofisher	Cat#31350010
Penicillin Streptomycin	Gibco	Cat#15140-122
FuGENE^®^ 6 Transfection Reagent	Promega	Cat#E2692
Polybrene Infection/Transfection Reagent	sigma	Cat#TR-1003-G
Cytofix^™^ Fixation Buffer (554655; BD Bioscience)	BD	Cat#554655
Sucrose	MP Biomedicals	Cat#152584
O.C.T. Compound	fisher healthcare	Cat#23-730-571
VWR^®^ Superfrost^®^ Plus Micro Slide, Premium	Avantor	Cat#48311-703
PBS, pH 7.4	ThermoFisher	Cat#10010031
Fluoromount G	Southern Biotech	Cat#0100-01
Coating buffer (pH = 9.5)	BD	Cat#51-2713KC
Stop Solution	invitrogen	Cat#SS04
normal mouse serum	Jackson immuno Research	Cat#015-000-120
normal rat serum	Jackson immuno Research	Cat#012-000-120
IL-2	R&D systems	Cat#401-ML
IL-12	PeproTech	Cat#210-12
IL-4	R&D Systems	Cat#404-ML
TGFβ1	R&D systems	Cat#7666-MB
IL-6	PeproTech	Cat#216-16
IL-1β	R&D systems	Cat#401-ML
Fixable Viability Dye eFluor^™^ 506	eBioscience	Cat# 65-0866-18
Fixable Viability Dye eFluor^™^ 780	eBioscience	Cat#65-0865-14
Dynabeads^™^ Mouse T-Activator CD3/CD28 for T cell Expansion and Activation	Thermofisher	Cat#11452D
UltraComp eBeads^™^ Compensation Bead	Thermofisher	Cat#01-2222-41
Phorbol 12-myristate 13-acetate	Sigma	Cat#P8139-5MG
Ionomycin calcium salt from Streptomyces conglobatus	Sigma	Cat#I0634-5MG
Monensin Solution (1000×)	eBioscience	Cat#00-4505-51
Critical commercial assays		
IgG1 Mouse Uncoated ELISA Kit with Plates	invitrogen	Cat#88-50410-86
IgG2c Mouse Uncoated ELISA Kit with Plates	invitrogen	Cat#88-50670-22
IgG (Total) Mouse Uncoated ELISA Kit with Plates	invitrogen	Cat#88-50400-86
miRNeasy micro kit	Qiagen	Cat#217084
DNase set	Qiagen	Cat#79254
QubitTM RNA High Sensitivity (HS), Assay Kit	ThermoFisher	Cat#Q10210
QubitTM dsDNA Broad Rang (BR) Assay Kit	ThermoFisher	Cat#Q32850
Dynabeads mRNA DIR ECT kit	Ambion Life Technologies	Cat#61012
EasySep^™^ Mouse Naive CD4^+^ T cell Isolation Kit	STEMCELL	Cat#19765
BD OptEIA^™^ Reagent Set Bt B	BD	Cat#550534
Foxp3 staining buffer set	eBioscience	Cat#00-5523-00
Deposited data		
RNA-Seq Raw and analyzed data	This paper	GSE247137
ChIP-Seq Raw and analyzed data	This paper	GSE247137
Experimental models: Organisms/strains		
Mouse: *Pou2af1*^−/−^,	This paper	N/A
Mouse: *Pou2af1*-HA-tdTomato	This paper	N/A
Mouse: *Bhlhe40*^−/−^	This paper	N/A
Mouse: *Bhlhe40*-V5	This paper	N/A
Mouse: *Bhlhe40*^fl/fl^-CD4Cre	This paper	Yu et al.^24^
Mouse: *Pou2af1^tm1a(KOMP)Wtsi^*	the University of California, Davis, KOMP Repository	www.mmrrc.org
Mouse: CD45.1^+^	NIAID-Taconic repository	Line 7
Mouse: *Rag1*^−/−^	NIAID-Taconic repository	Line 146
Mouse: *TCRα*^−/−^	NIAID-Taconic repository	Line 98
Mouse: Cas9	JAXSON	JAX 026179
Mouse: OT-II	NIAID-Taconic repository	Line 187
FlowJo V10	Tree Star	https://www.flowjo.com
GraphPad Prism 9	GraphPad	https://www.graphpad.com
Imaris	Bitplane	https://imaris.oxinst.com

## Highlights

- Transcription factor Bhlhe40 inhibits Pou2af1 expression in CD4 T cells
- The Bhlhe40-Pou2af1 axis regulates the maturation of follicular T cells
- The Bhlhe40-Pou2af1 axis does not affect initial Bcl6 induction
- Pou2af1 promotes optimal CXCR5 expression and cell migration into germinal centers
