# Supplementary material for: Optimal CXCR5 expression during Tfh maturation involves the Bhlhe40-Pou2af1 axis
Source: Cell Rep. Author manuscript; Available in PMC 2025 Dec 28. (PMC12744931; doi:10.1016/j.celrep.2025.116470)
Supplement: 1 [file NIHMS2125807-supplement-1.pdf]

Cell Reports, Volume *44*

Supplemental information

Optimal CXCR5 expression during Tfh maturation involves the Bhlhe40-Pou2af1 axis

Xiaoliang Zhu, Xi Chen, Yaqiang Cao, Chengyu Liu, Zoey J. Kline, Gangqing Hu, Sundar Ganesan, Tibor Z. Veres, Difeng Fang, Shuai Liu, Danping Wei, Hirofumi Shibata, Dominic P. Golec, Hyunwoo Chung, Ronald N. Germain, Pamela L. Schwartzberg, Keji Zhao, and Jinfang Zhu

Fig.S1

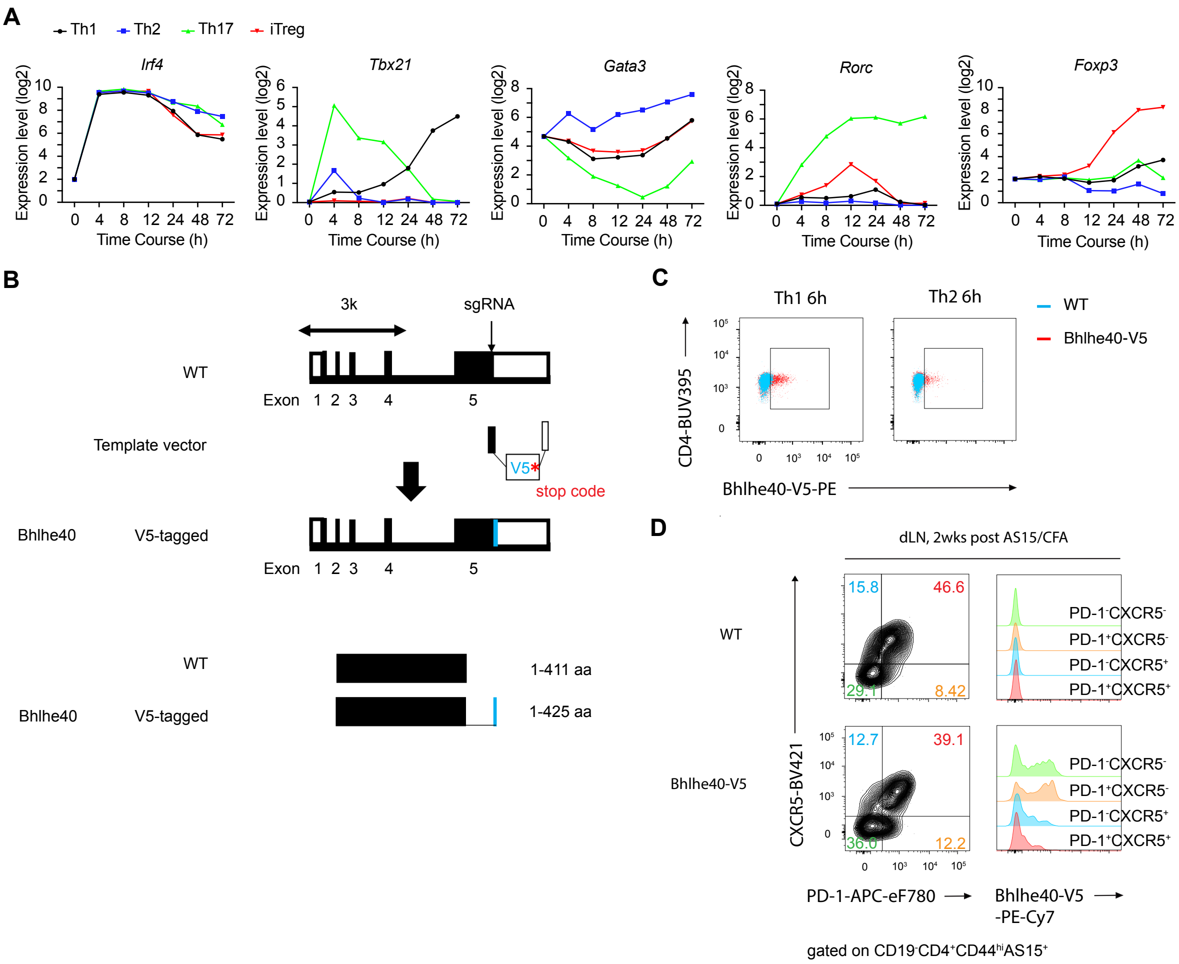
A
Th1
Th2
Th17
iTreg
Irf4
Tbx21
Gata3
Rorc
Foxp3
Expression level (log2)
Time Course (h)
B
3k
sgRNA
WT
Exon 1 2 3 4 5
Template vector
V5
stop code
Bhlhe40 V5-tagged
WT
1-411 aa
Bhlhe40 V5-tagged
1-425 aa
C
Th1 6h
Th2 6h
WT
Bhlhe40-V5
CD4-BUV395
Bhlhe40-V5-PE
D
dLN, 2wks post AS15/CFA
WT
15.8
46.6
29.1
8.42
PD-1-CXCR5-
PD-1+CXCR5-
PD-1-CXCR5+
PD-1+CXCR5+
Bhlhe40-V5
12.7
39.1
36.0
12.2
CXCR5-BV421
PD-1-APC-eF780
Bhlhe40-V5 -PE-Cy7
gated on CD19-CD4+CD44hiAS15+

Fig. S1 (related to Fig. 1) Confirmation of key genes expressed in conventional CD4 T cells, generation of Bhlhe40-V5 mouse strain, and its usage in confirming *Bhlhe40* expression at protein level.

(**A**) RNA-Seq data were re-analyzed for indicated genes expression during priming of Th1, Th2, Th17 and iTreg in vitro.
(**B**) Strategy of generating Bhlhe40 V5-tagged mice.
(**C**) Naïve CD4 T cells were purified from Bhlhe40-V5 mice and primed under Th1 and Th2 conditions. Bhlhe40 expression was assessed by anti-V5 staining after TCR stimulation for 6 hours.
(**D**) WT and Bhlhe40-V5 mice were immunized (s.c.) with AS15/CFA for 2 weeks, and the expression of Bhlhe40 by AS15-specific effective CD4 T cells from inguinal lymph nodes divided into four populations (PD-1^-CXCR5^-, PD-1^+CXCR5^-, PD-1^-CXCR5^+, PD-1^+CXCR5^+) was assessed by flow cytometry.
Data are representative of two (C and D) independent experiments.

Fig.S2

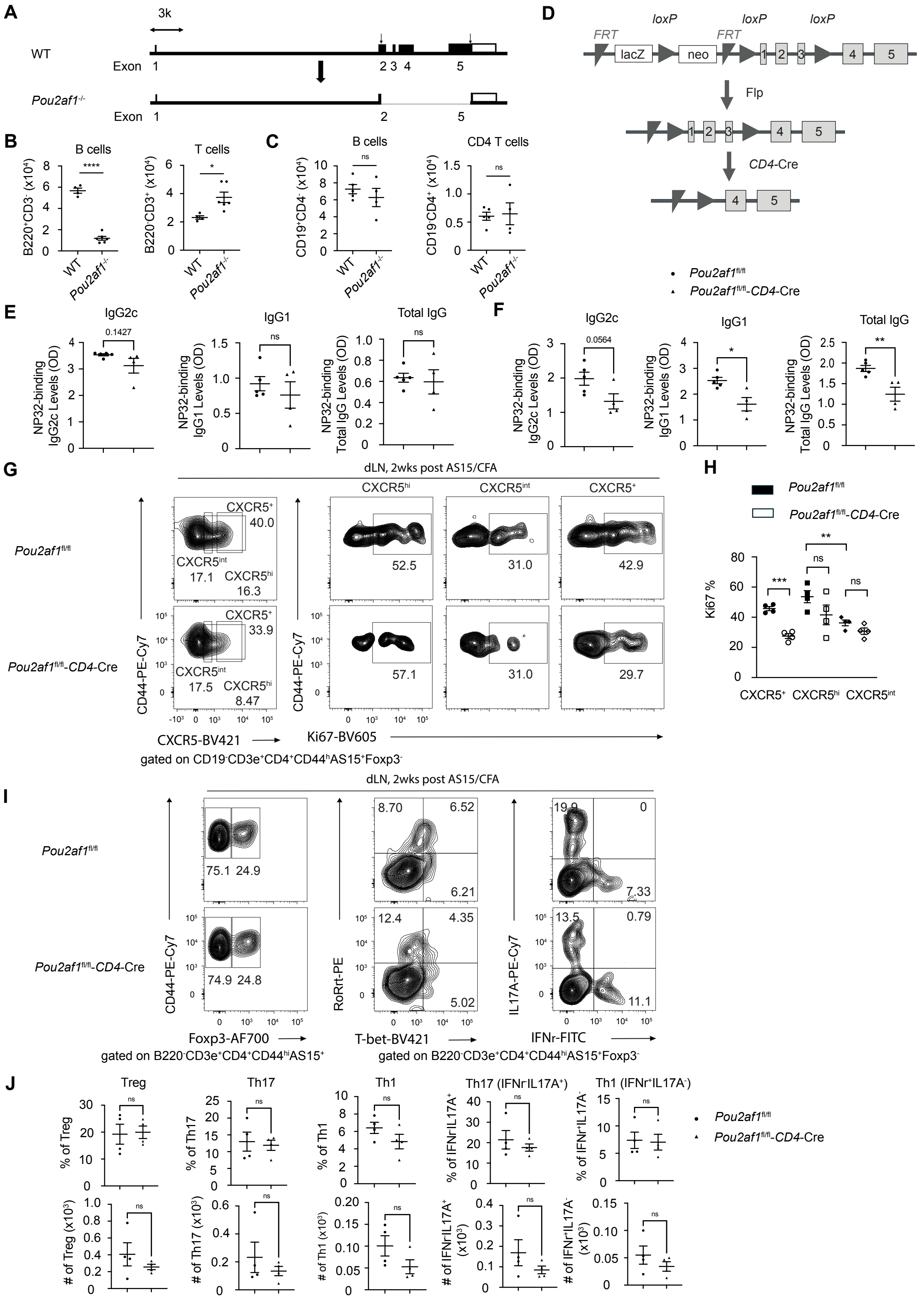

A
3k
WT
Exon 1 2 3 4 5
Pou2af1-/-
Exon 1 2 5
B
B cells
T cells
B220+CD3- (x10⁴)
B220-CD3+ (x10⁴)
WT
Pou2af1-/-
C
B cells
CD4 T cells
CD19+CD4- (x10⁴)
CD19-CD4+ (x10⁴)
D
FRT
loxP
lacZ
neo
Flp
CD4-Cre
Pou2af1fl/fl
Pou2af1fl/fl-CD4-Cre
E
IgG2c
IgG1
Total IgG
NP32-binding IgG2c Levels (OD)
NP32-binding IgG1 Levels (OD)
NP32-binding Total IgG Levels (OD)
F
G
dLN, 2wks post AS15/CFA
CXCR5hi
CXCR5int
CXCR5+
CD44-PE-Cy7
CXCR5-BV421
Ki67-BV605
gated on CD19-CD3e+CD4+CD44hiAS15+Foxp3-
H
Ki67 %
I
Foxp3-AF700
T-bet-BV421
RoRrt-PE
IL17A-PE-Cy7
IFNr-FITC
gated on B220-CD3e+CD4+CD44hiAS15+
gated on B220-CD3e+CD4+CD44hiAS15+Foxp3-
J
Treg
Th17
Th1
Th17 (IFNr-IL17A+)
Th1 (IFNr+IL17A-)
% of Treg
% of Th17
% of Th1
# of Treg (x10³)
# of Th17 (x10³)
# of Th1 (x10³)

Fig. S2 (related to Fig. 2) Pou2af1 is required for Tfh cell differentiation and B cell responses but has no effect on the differentiation of other effector CD4 T cells.
(**A**) Strategy for generating *Pou2af1*$^{-/-}$ mice via CRISPR-Cas9 technology.
(**B**) Summary of cell number difference in B cells and T cells comparing WT (*n*=4) and *Pou2af1*$^{-/-}$ (*n*=6) mice with the same amount of blood volume in Fig.2A.
(**C**) Summary of cell number difference in B cells and CD4 T cells comparing *Tcra*$^{-/-}$ recipients adoptively transferred with WT (*n*=5) or *Pou2af1*$^{-/-}$ (*n*=4) with the same amount of blood volume in Fig.2E.
(**D**) Schematic view of generation of *Pou2af1*$^{fl/fl}$-CD4-Cre mouse strain. The KO-first allele contains a trapping cassette with a lacZ reporter and a floxed promoter-driven neo cassette inserted into promoter of *Pou2af1* gene, disrupting *Pou2af1* gene function. Flpe converts the KO-first allele to a conditional allele, restoring *Pou2af1* gene activity. *CD4*-Cre deletes the floxed exon 1-3 to generate T cell–specific deletion of the *Pou2af1* gene.
(**E**) ELISA of NP-specific antibodies in serum 2 wks after NP-KLH/CFA immunization.
(**F**) ELISA of NP-specific antibodies in serum 6 wks after NP-KLH/CFA immunization.
(**G-J**) *Pou2af1*$^{fl/fl}$ and *Pou2af1*$^{fl/fl}$-CD4Cre mice were immunized (s.c.) with AS15/CFA for 2 weeks, and cells from inguinal lymph nodes were harvested and analyzed. (G) AS15-specific-non-Treg (Foxp3^{neg}) cells in inguinal lymph nodes were further gated for three populations (CXCR5^{+}, CXCR5^{hi} and CXCR5^{low}) to analyze Ki67 expression by FACS. (H) Summary of percentage of Ki67 in three populations (CXCR5^{+}, CXCR5^{hi} and CXCR5^{low}) of (G) from *Pou2af1*$^{fl/fl}$ group (n=4) and *Pou2af1*$^{fl/fl}$-CD4Cre group (n=4). (I) Treg cells (Foxp3^{+}), Th1 cells (Foxp3^{-}T-bet^{+}RORγt^{-}), and Th17 cells (Foxp3^{-}T-bet^{-}RORγt^{+}) in inguinal lymph nodes were analyzed by FACS. (J) Summary of percentage of Treg cells, Th1 cells and Th17 cells from *Pou2af1*$^{fl/fl}$ group (n=4) and *Pou2af1*$^{fl/fl}$-CD4Cre group (n=4) in (I).
* $p < 0.05$, ** $p < 0.01$, *** $p < 0.001$, **** $p < 0.0001$, Student's *t*-test. Error bars indicate SEM. Data are representative of more than three (B) and two (C, E-J) independent experiments.

Fig.S3

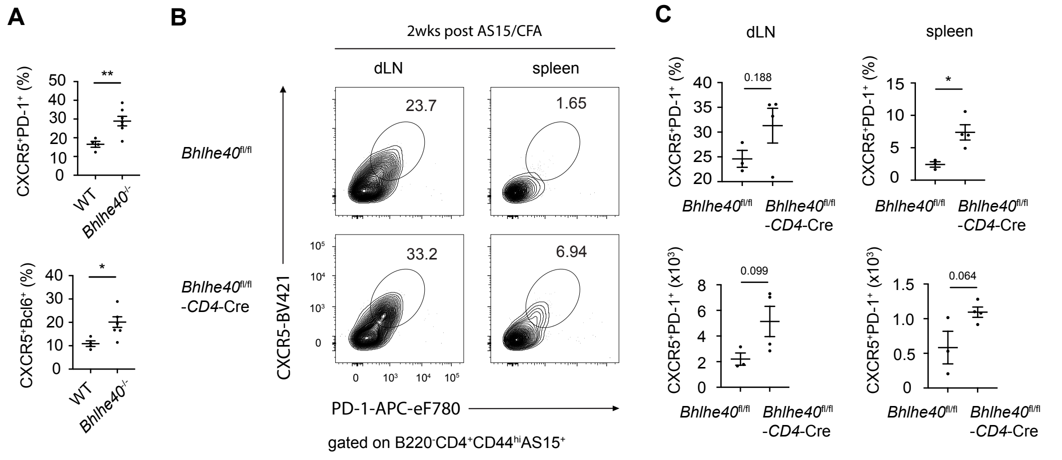
A
CXCR5+PD-1+ (%)
**
WT
Bhlhe40-/-
CXCR5+Bcl6+ (%)
*
B
2wks post AS15/CFA
dLN
spleen
Bhlhe40fl/fl
Bhlhe40fl/fl
-CD4-Cre
23.7
1.65
33.2
6.94
CXCR5-BV421
PD-1-APC-eF780
gated on B220-CD4+CD44hiAS15+
C
dLN
spleen
CXCR5+PD-1+ (%)
0.188
*
CXCR5+PD-1+ (x10³)
0.099
0.064
Bhlhe40fl/fl
Bhlhe40fl/fl
-CD4-Cre

Fig. S3. (related to Fig. 3) Bhlhe40 represses Tfh cell differentiation.
(**A**) Summary of percentage difference in Tfh cells (PD-1^{+}CXCR5^{+} or Bcl6^{+}CXCR5^{+}) between WT (n=4) and *Bhlhe40*$^{-/-}$ (n=7) in the spleen in Fig. 3A.
(**B**) WT (n=3) and *Bhlhe40*$^{fl/fl}$-CD4Cre (n=4) mice were immunized (s.c.) with AS15/CFA for 2 weeks, and AS15-specific CD4 T cells from inguinal lymph nodes and spleen were analyzed for Tfh (PD-1^{+}CXCR5^{+} or Bcl6^{+}CXCR5^{+}) cells by flow cytometry.
(**C**) Summary of percentage difference in Tfh cells between WT and *Bhlhe40*$^{fl/fl}$-CD4Cre in lymph nodes and spleen in (B).
* $p < 0.05$, ** $p < 0.01$, *** $p < 0.001$, **** $p < 0.0001$, Student's *t*-test. Error bars indicate SEM. Data are representative of two (A-C) independent experiments.

Fig.S4

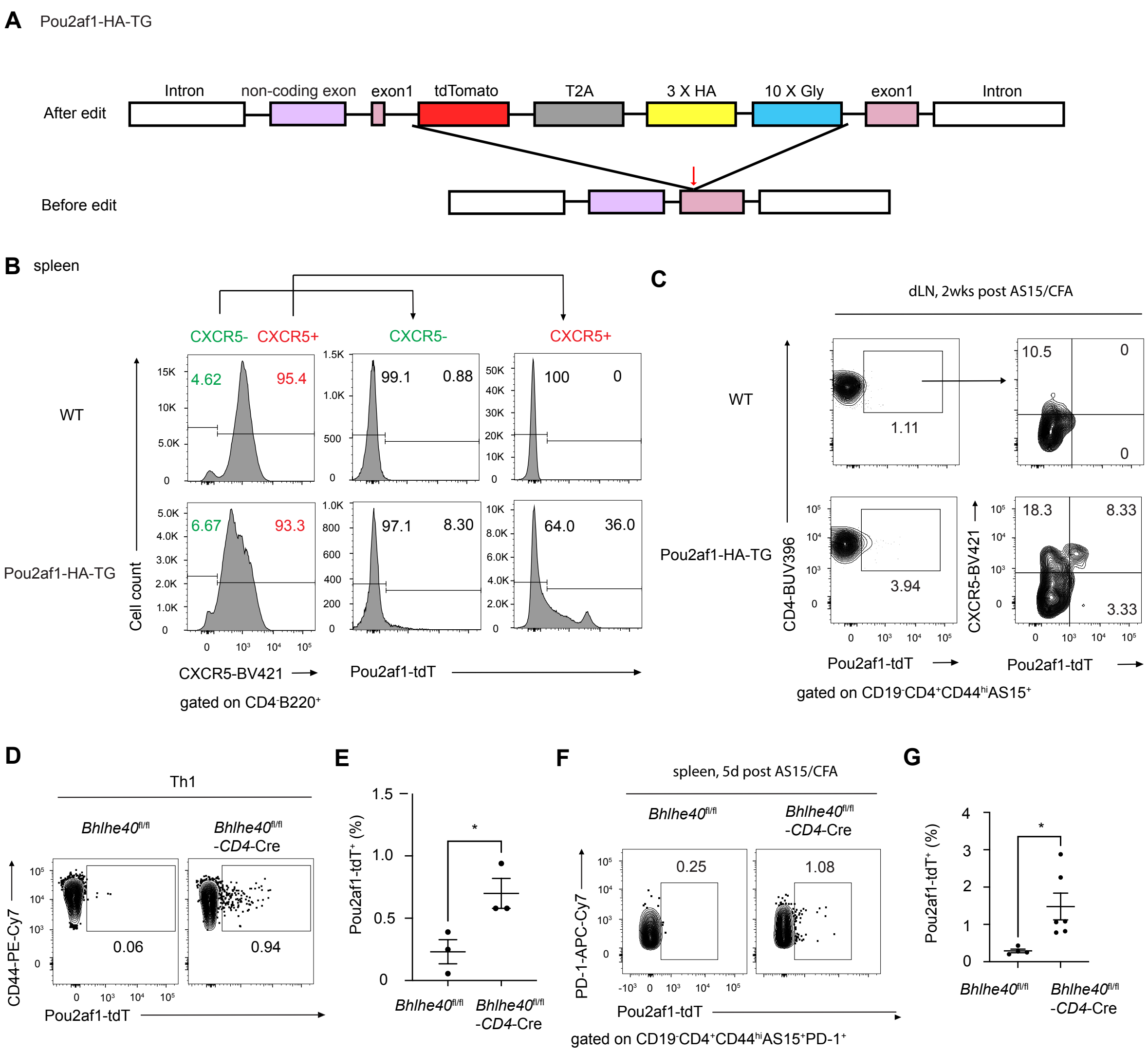

A Pou2af1-HA-TG
After edit
Intron
non-coding exon
exon1
tdTomato
T2A
3 X HA
10 X Gly
exon1
Intron
Before edit
B spleen
CXCR5- CXCR5+
CXCR5-
CXCR5+
WT
Pou2af1-HA-TG
Cell count
4.62
95.4
99.1
0.88
100
0
6.67
93.3
97.1
8.30
64.0
36.0
CXCR5-BV421
Pou2af1-tdT
gated on CD4-B220+
C
dLN, 2wks post AS15/CFA
WT
Pou2af1-HA-TG
CD4-BUV396
1.11
3.94
10.5
18.3
8.33
3.33
CXCR5-BV421
Pou2af1-tdT
gated on CD19-CD4+CD44hiAS15+
D
Th1
Bhlhe40fl/fl
Bhlhe40fl/fl -CD4-Cre
CD44-PE-Cy7
0.06
0.94
Pou2af1-tdT
E
Pou2af1-tdT+ (%)
*
Bhlhe40fl/fl
Bhlhe40fl/fl -CD4-Cre
F
spleen, 5d post AS15/CFA
Bhlhe40fl/fl
Bhlhe40fl/fl -CD4-Cre
PD-1-APC-Cy7
0.25
1.08
Pou2af1-tdT
gated on CD19-CD4+CD44hiAS15+PD-1+
G
Pou2af1-tdT+ (%)
*
Bhlhe40fl/fl
Bhlhe40fl/fl -CD4-Cre

Fig. S4 (related to Fig. 4) Generation of Pou2af1-HA-TG mouse strain and its usage in confirming the relationship between Pou2af1 and CXCR5 or Bhlhe40.

(**A**) Strategy of generating Pou2af1-HA-TG mouse strain via CRISPR-Cas9. tdTomato (red box) and three repeats of HA (yellow box) were linked to the N-terminal of Pou2af1.

(**B**) The expression of CXCR5 by B cells ($CD4^{-}B220^{+}$) from WT or Pou2af1-HA-TG mice was analyzed. $CXCR5^{-}$ and $CXCR5^{+}$ populations were further assessed for Pou2af1-tdTomato (Pou2af1-tdT) expression.

(**C**)WT and Pou2af1-HA-TG mice were immunized (s.c.) with AS15/CFA for 2 weeks, and AS15-specific CD4 T cells ($CD19\text{-}CD4^{+}CD44^{hi}AS15^{+}$) from inguinal lymph nodes and spleen were analyzed.

(**D**) WT or *Bhlhe40*$^{fl/fl}$-CD4Cre mice were crossed with Pou2af1-HA-TG mouse to generate Pou2af1-tdTomato-bearing WT (*n*=3) or *Bhlhe40*$^{fl/fl}$-CD4Cre (*n*=3) reporter mice. Then naïve CD4 T cells were purified from such reporter mice with indicated genotypes and primed under Th1 conditions. Pou2af1 expression in Th1 cells were analyzed by flow cytometry.

(**E**) Summary of percentage of $Pou2af1^{+}$ Th1 cells in (D).

(**F**) Pou2af1-tdTomato-bearing *Bhlhe40*$^{fl/fl}$ (n=4), and Pou2af1-tdTomato-bearing *Bhlhe40*$^{fl/fl}$-CD4-Cre (n=6) reporter mice were immunized (s.c.) with AS15/CFA for 5d, and AS15-specific effective CD4 T cells from spleen were gated on PD-1^{+} and analyzed for Pou2af1-tdTomato expression by flow cytometry.

(**G**) Summary of percentage difference of Pou2af1-tdTomato between *Bhlhe40*$^{fl/fl}$ and *Bhlhe40*$^{fl/fl}$-CD4Cre in vivo.

* $p < 0.05$, ** $p < 0.01$, *** $p < 0.001$, **** $p < 0.0001$, Student's *t*-test. Error bars indicate SEM. Data are representative of two (B-G) independent experiments.

Fig.S5

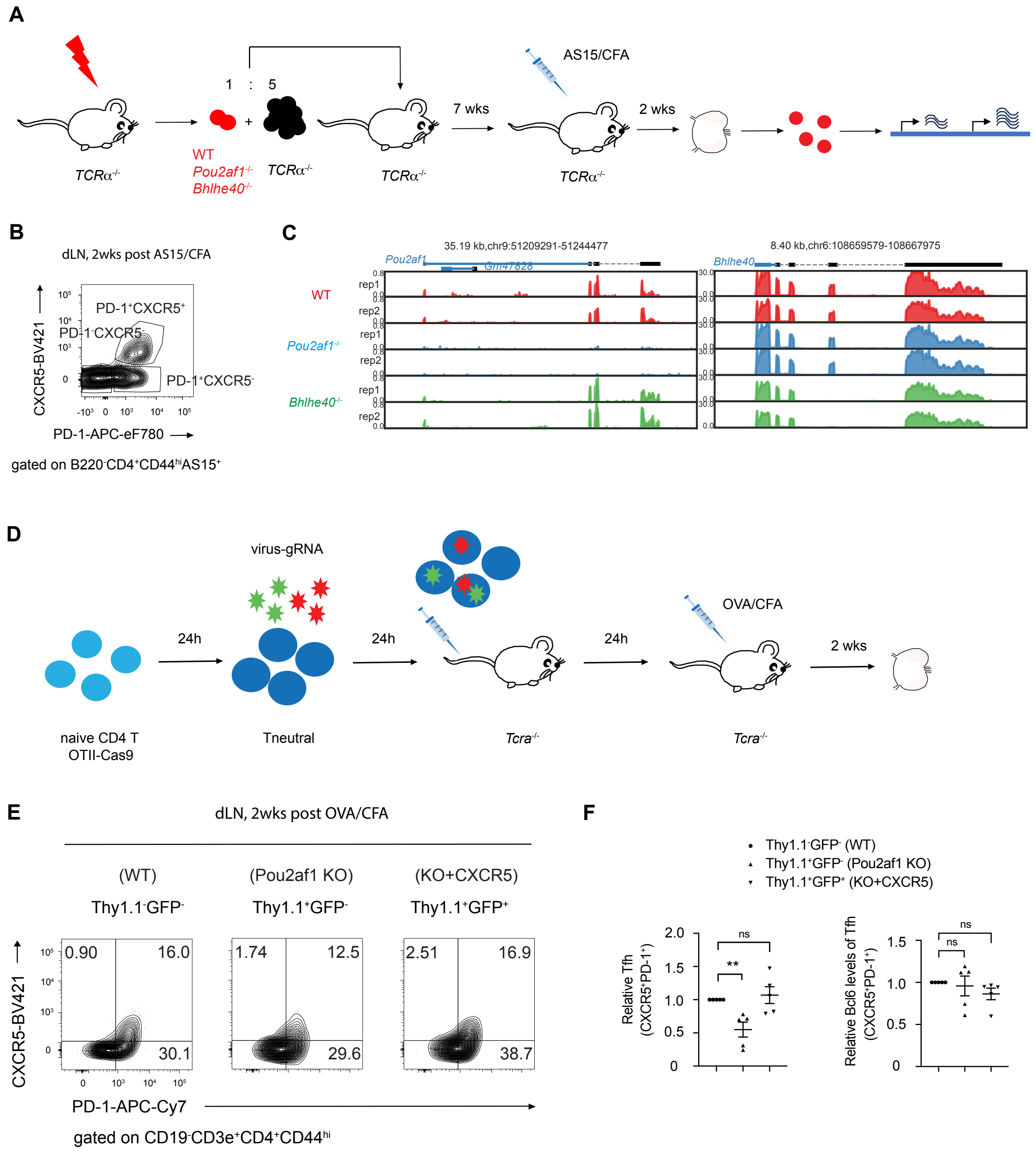
A
1 : 5
AS15/CFA
7 wks
2 wks
TCRα-/-
WT
Pou2af1-/-
Bhlhe40-/-
TCRα-/-
TCRα-/-
TCRα-/-
B
dLN, 2wks post AS15/CFA
PD-1+CXCR5+
PD-1-CXCR5-
PD-1+CXCR5-
CXCR5-BV421
PD-1-APC-eF780
gated on B220-CD4+CD44hiAS15+
C
35.19 kb,chr9:51209291-51244477
8.40 kb,chr6:108659579-108667975
Pou2af1
Gm47828
Bhlhe40
WT
Pou2af1-/-
Bhlhe40-/-
rep1
rep2
D
virus-gRNA
24h
24h
24h
OVA/CFA
2 wks
naive CD4 T
OTII-Cas9
Tneutral
Tcra-/-
Tcra-/-
E
dLN, 2wks post OVA/CFA
(WT)
(Pou2af1 KO)
(KO+CXCR5)
Thy1.1-GFP-
Thy1.1+GFP-
Thy1.1+GFP+
0.90
16.0
30.1
1.74
12.5
29.6
2.51
16.9
38.7
CXCR5-BV421
PD-1-APC-Cy7
gated on CD19-CD3e+CD4+CD44hi
F
Thy1.1-GFP- (WT)
Thy1.1+GFP- (Pou2af1 KO)
Thy1.1+GFP+ (KO+CXCR5)
Relative Tfh
(CXCR5+PD-1+)
Relative Bcl6 levels of Tfh
(CXCR5+PD-1+)
ns
**
ns
ns

Fig. S5 (related to Fig. 5) Pou2af1 and Bhlhe40 regulate Tfh differentiation in cell intrinsic manner.

(**A**) Experimental procedure of immunizing bone marrow chimeras *Tcra*$^{-/-}$ mice, received adoptively transferred bone marrow mixture of *Tcra*$^{-/-}$ with WT, *Pou2af1*$^{-/-}$ or *Bhlhe40*$^{-/-}$, with AS15/CFA followed by RNA-Seq analysis.

(**B**) AS15-specific CD4 T cells (CD19^{-}CD4^{+}CD44^{hi}AS15^{+}) were sorted for three populations (PD-1^{-}CXCR5^{-}, PD-1^{+}CXCR5^{-}, PD-1^{+}CXCR5^{+}) from draining lymph nodes and used for RNA-Seq analysis.

(**C**) Biological duplicates of the RNA-Seq data were shown to confirm the genotype background of the materials.

(**D**) Experimental procedure of immunizing *Tcra*$^{-/-}$ mice received transfer of viral infected cells generated from OTII-Cas9 strain with OVA/CFA.

(**E**) Tneutral cells infected with mPou2af1KO-gRNA3-Thy1.1 virus and MSCV-CXCR5-GFP virus were adoptive transferred into *Tcra*$^{-/-}$ mice as shown in D, and effector CD4 T cells from inguinal lymph nodes were analyzed for Tfh (CXCR5^{+}PD-1^{+}) cells in three populations (WT: Thy1.1^{-}GFP^{-}; Pou2af1 KO: Thy1.1^{+}GFP^{-}; KO+CXCR5: Thy1.1^{+}GFP$^{+)}$.

(**F**) Summary of percentage difference of relative Tfh cells (CXCR5^{+}PD-1^{+}) and relative Bcl6 expression levels in Tfh cells in three indicated populations within same mice by normalizing WT (Thy1.1^{-}GFP^{-}) as 1. n=5.

* $p < 0.05$, ** $p < 0.01$, *** $p < 0.001$, **** $p < 0.0001$, Student's *t*-test. Error bars indicate SEM. Samples are in biological duplicates (B) and data (E-F) are representative of two independent experiments.

Fig.S6

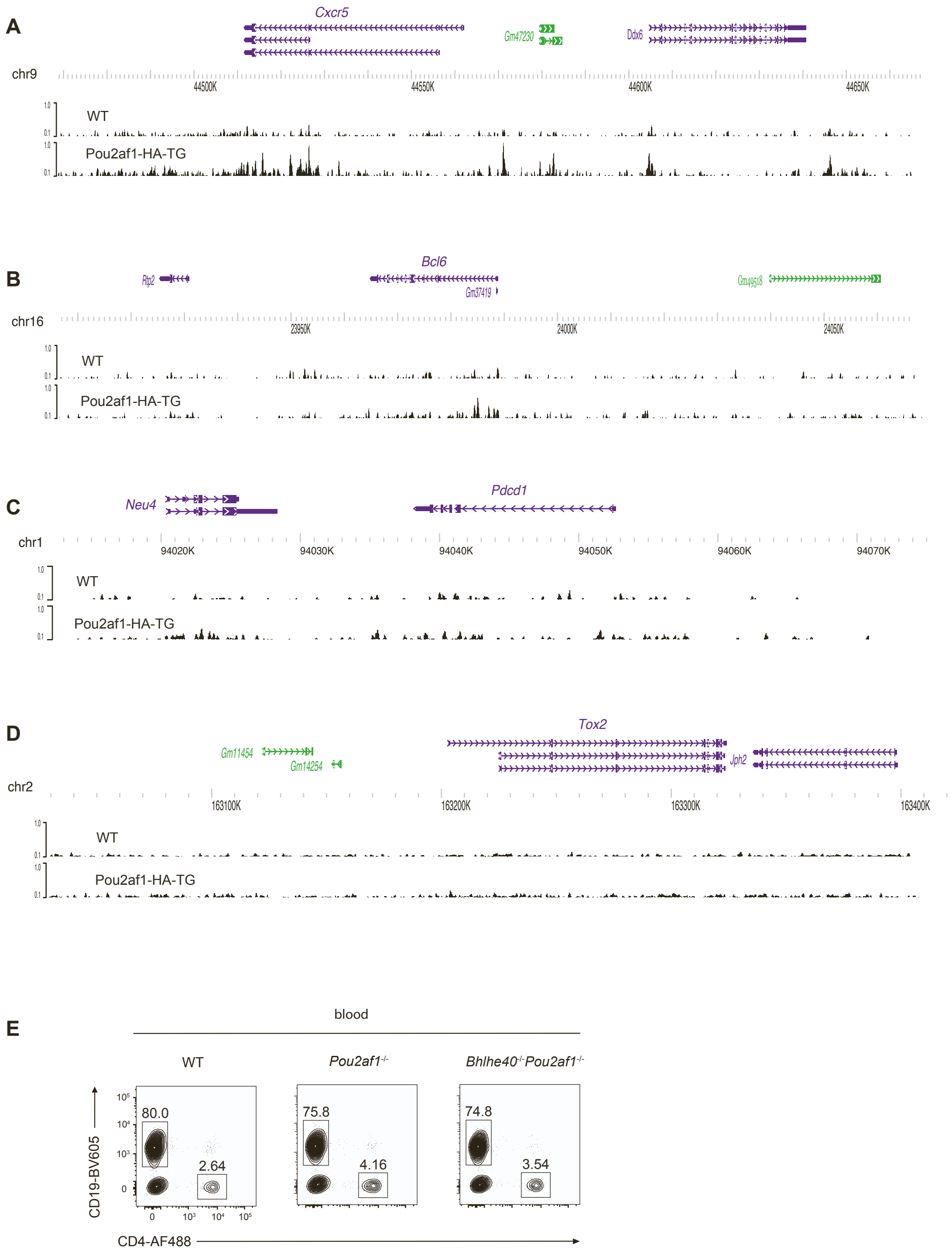
A
Cxcr5
Gm47230
Ddx6
chr9
44500K
44550K
44600K
44650K
WT
Pou2af1-HA-TG
B
Rtp2
Bcl6
Gm37419
Gm49518
chr16
23950K
24000K
24050K
WT
Pou2af1-HA-TG
C
Neu4
Pdcd1
chr1
94020K
94030K
94040K
94050K
94060K
94070K
WT
Pou2af1-HA-TG
D
Gm11454
Gm14254
Tox2
Jph2
chr2
163100K
163200K
163300K
163400K
WT
Pou2af1-HA-TG
E
blood
WT
Pou2af1-/-
Bhlhe40-/-Pou2af1-/-
80.0
2.64
75.8
4.16
74.8
3.54
CD19-BV605
CD4-AF488

Fig. S6 (related to Fig. 5 and Fig. 6) Differential Pou2af1 binding patterns to the Tfh-related genes, *Cxcr5*, *Bcl6*, *Pdcd1* and *Tox2*; analysis of bone marrow reconstitution of the chimeric mice.
(A-D) Splenic B cells were sorted from WT or Pou2af1-HA-TG mice, and anti-HA was used for ChIP-Seq analysis for Pou2af1 binding in the genome. Genome browser view of Pou2af1 binding at the gene locus of *Cxcr5* (A), *Bcl6* (B), *Pdcd1* (C) and *Tox2* (D), respectively.
(**E**) B cells and CD4 T cells from indicated mice groups were assessed by flow cytometry in bone marrow chimeras shown in Fig. 6C.

Fig.S7

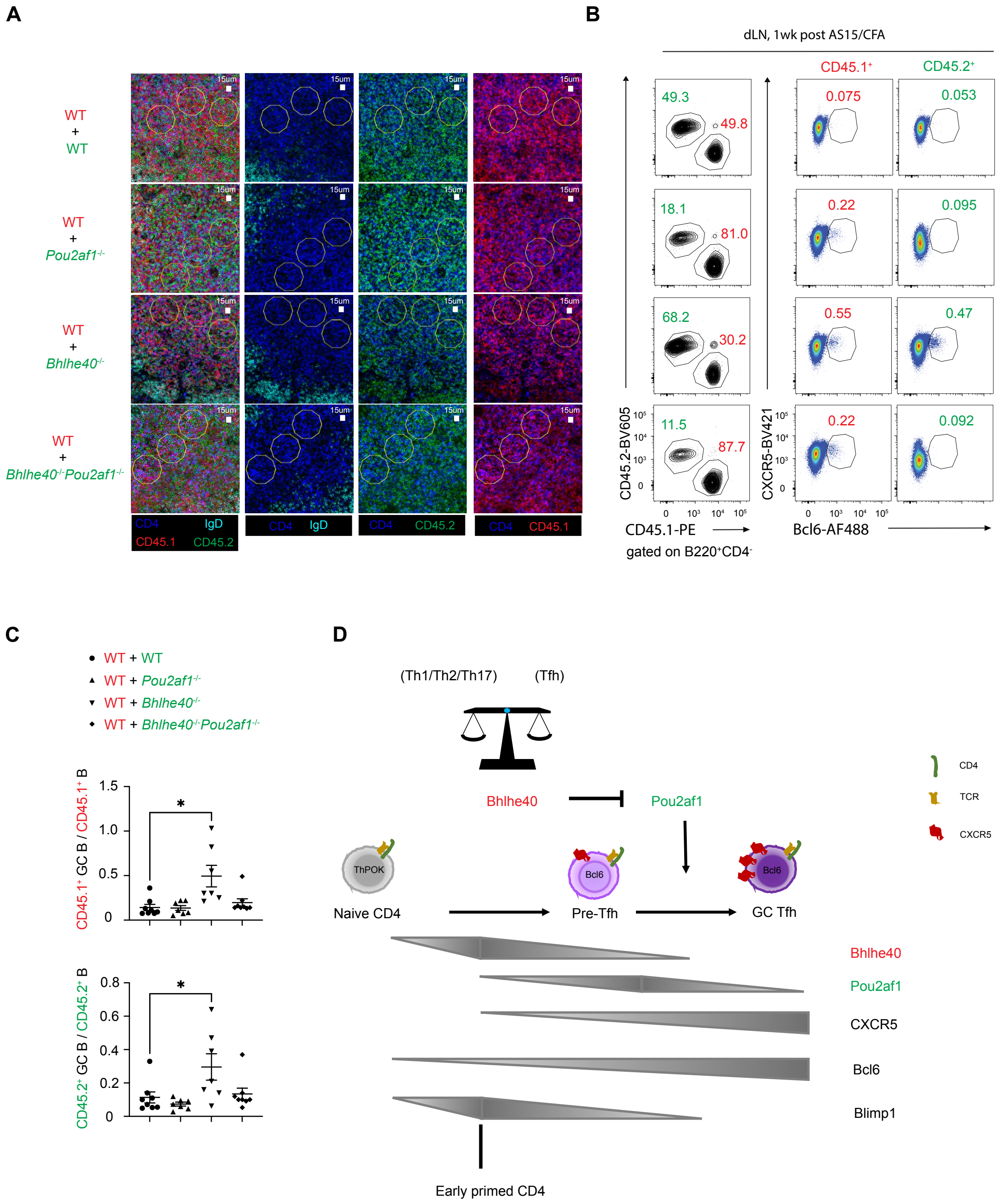
A
WT + WT
WT + Pou2af1-/-
WT + Bhlhe40-/-
WT + Bhlhe40-/-Pou2af1-/-
15um
CD4 IgD CD45.1 CD45.2
CD4 IgD
CD4 CD45.2
CD4 CD45.1
B
dLN, 1wk post AS15/CFA
CD45.1+
CD45.2+
49.3
49.8
0.075
0.053
18.1
81.0
0.22
0.095
68.2
30.2
0.55
0.47
11.5
87.7
0.22
0.092
CD45.2-BV605
CD45.1-PE
gated on B220+CD4-
CXCR5-BV421
Bcl6-AF488
C
WT + WT
WT + Pou2af1-/-
WT + Bhlhe40-/-
WT + Bhlhe40-/-Pou2af1-/-
CD45.1+ GC B / CD45.1+ B
CD45.2+ GC B / CD45.2+ B
D
(Th1/Th2/Th17)
(Tfh)
Bhlhe40
Pou2af1
CD4
TCR
CXCR5
ThPOK
Bcl6
Naive CD4
Pre-Tfh
GC Tfh
Bhlhe40
Pou2af1
CXCR5
Bcl6
Blimp1
Early primed CD4

Fig. S7 (related to Fig. 7) Regulation of optimal CXCR5 level through Bhlhe40-Pou2af1 axis during Tfh cell maturation.
(**A**) Inguinal lymph nodes from Fig. 7A were fixed and stained for confocal image. Anti-CD4 (blue) and anti-IgD (cyan) were used to distinguish T cell area and B cell area. Three T cell areas (yellow decagon) were chosen randomly for further analysis. Anti-CD45.2 (green) as used to show different indicated genotyped adoptive cells, and anti-CD45.1 (red) was used to show WT adoptive cells from the same mouse. $CD4^+CD45.2^+$ indicated different genotype background adoptive CD4 T cells. $CD4^+CD45.1^+$ indicated WT adoptive CD4 T cells.
(**B**) B cells ($B220^+CD4^-$) from inguinal lymph nodes from Fig. 7A were assessed by flow cytometry in four different groups as (A). And GC B cells ($Bcl6^+CXCR5^+$) were further gated to show their distribution in different groups.
(**C**) Summary of WT ($CD45.1^+CD45.2^-$) GC B cells or indicated genotyped ($CD45.1^-CD45.2^+$) GC B cells percentage difference within their own total B cells in four different groups, n=7-8.
(**D**) A model for Bhlhe40-Pou2af1 axis in regulating Tfh cell maturation by optimizing CXCR5 expression levels.
* $p < 0.05$, ** $p < 0.01$, *** $p < 0.001$, **** $p < 0.0001$, Student's *t*-test. Error bars indicate SEM. Data are representative of two independent experiments (A-C).
